# Cell clusters softening triggers collective cell migration in vivo

**DOI:** 10.1038/s41563-022-01323-0

**Published:** 2022-08-15

**Authors:** Cristian L. Marchant, Abdul N. Malmi-Kakkada, Jaime A. Espina, Elias H. Barriga

**Affiliations:** 1 Mechanisms of Morphogenesis Laboratory, Gulbenkian Institute of Science (IGC), Oeiras, Portugal; 2 Computational Biological Physics Laboratory, Department of Chemistry and Physics, Augusta University, Augusta, GA USA

**Keywords:** Collective cell migration, Developmental biology

## Abstract

Embryogenesis, tissue repair and cancer metastasis rely on collective cell migration. In vitro studies propose that cells are stiffer while migrating in stiff substrates, but softer when plated in compliant surfaces which are typically considered as non-permissive for migration. Here we show that cells within clusters from embryonic tissue dynamically decrease their stiffness in response to the temporal stiffening of their native substrate to initiate collective cell migration. Molecular and mechanical perturbations of embryonic tissues reveal that this unexpected mechanical response involves a mechanosensitive pathway relying on Piezo1-mediated microtubule deacetylation. We further show that decreasing microtubule acetylation and consequently cluster stiffness is sufficient to trigger collective cell migration in soft non-permissive substrates. This suggests that reaching an optimal cluster-to-substrate stiffness ratio is essential to trigger the onset of this collective process. Overall, these in vivo findings challenge the current understanding of collective cell migration and its physiological and pathological roles.

## Abstract (web summary)

Collective cell migration in embryonic tissues is triggered by cell softening due to a microtubule deacetylation pathway involving the mechanosensitive ion channel Piezo1.

## Main

A wide variety of biological processes such as embryogenesis, tissue repair and cancer metastasis rely on the migration of individual cells or on the coordinated movement of cell clusters via a process named collective cell migration (CCM)^1,2^. The interaction between migrating cells, or clusters, and the mechanical properties of their substrates have been widely studied in vitro^3^ and more recently in vivo^4,5^. Thus, it is well established that stiffer substrates favour individual and CCM and in vitro evidence support the idea that the elastic properties of migrating cells and their environment are directly dependent^6,7^. However, recent in silico and in vitro evidence indicates that this may not be the case when cells are plated on compliant surfaces, such as those observed in some in vivo environments^8,9^. Hence, whether and how cells or group of cells that migrate in a dynamic and convoluted in vivo environment adjust their mechanical properties in relation to the substrate remains unclear.

Here we study the mechanical interplay between migrating cell clusters and their native substrate in vivo using as a model the collective migration of the *Xenopus laevis* cephalic neural crest (NC), a mechanosensitive embryonic cell population whose invasive ability has been likened to cancer^10^. We find that cells in these migrating clusters dynamically decrease their stiffness in response to substrate stiffening thus triggering CCM. This behaviour is mediated by a mechanosensitive pathway involving Piezo1-mediated microtubule deacetylation. We further show that cell clusters can be stimulated to migrate by biochemically decreasing microtubule acetylation, even when in soft substrates, suggesting that an optimal cluster-to-substrate stiffness ratio is involved in CCM onset.

## Reduction of cluster stiffness at the onset of CCM in vivo

**Fig. 1:**
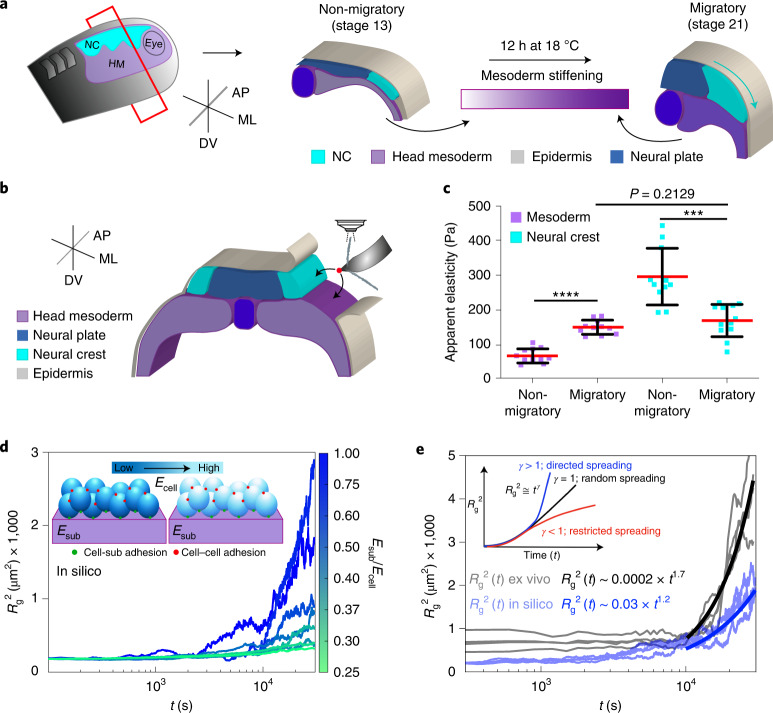
NC cells reduce their stiffness at the onset of CCM in vivo. **a**, Diagram represents a cross-section of a *X. laevis* embryo showing the development of NC (HM, head mesoderm; ML, mediolateral; AP, anteroposterior; DV, dorso-ventral). Cephalic or cranial NC originates from ectoderm at the border of the neural plate and their CCM is triggered by stiffening of the head mesoderm, the NC migratory substrate. **b**, Schematic showing the regions measured by iAFM in wild-type or treated embryos, black arrows point to the recorded regions. **c**, Spread of data for each condition as stated in the figure, red lines represent the mean and whiskers the standard deviation (s.d.) (two-tailed *t*-test ****P < 0.0001, ***P = 0.0004, CI = 95%, *n*_non-migratory mesoderm_ = 12, *n*_migratory mesoderm_ = 11, *n*_non-migratory NC_ = 11, *n*_migratory NC_ = 12 embryos; 64 indentations were performed per embryo). **d**, Top left inset in the graph correspond to a simplified representation of our mathematical model used to obtain *R*_g_^2^. Briefly, the behaviour of cells (which are connected between them, red dots, and connected to the substrate, green dots) was simulated at varying stiffness values when spreading on a substrate of fixed stiffness (magenta). The result of these simulations is shown as a chart were *R*_g_^2^ calculations under different *E*_sub_*/E*_cell_ regimes are presented, lines represent *R*_g_^2^ over time. **e**, Comparison of in silico (shaded blue lines) and ex vivo (shaded black lines) *R*_g_^2^ calculations (*E*_sub_*/E*_cell_ > 1 in both conditions). Inset showing potential outcomes of *R*_g_^2^ ≅ *t*^*γ*^ as an output of cell directionality; directionality extracted from in silico (solid blue lines) and ex vivo (solid black lines) *R*_g_^2^ ≅ *t*^*γ*^ are shown. Source data

**Extended Data Fig. 1:**
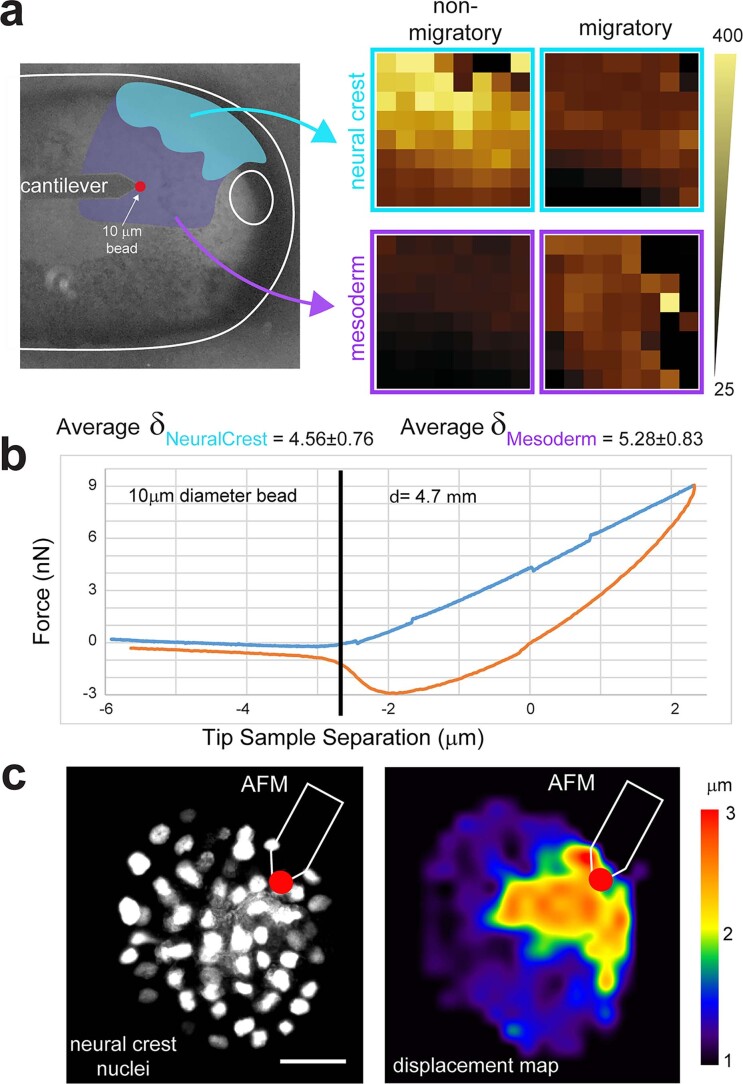
*in vivo* Atomic Force Microscopy experiments setup. (**a**) Image of the AFM cantilever position relative to the neural crest (cyan) and head mesoderm (magenta). A heat map showing measurements acquired in an 8×8 grid with 6.25 μm x/y space resolution is shown; this grid depicts the spread of data found in the tissues we measured. All measurements were recorded by using a cantilever carrying a 10 μm diameter bead as a tip. Each data point in our charts presented in the main figures represents an embryo from which the median resulting from our 8×8 grids was calculated. (**b**) Representative example of a force-distance curve obtained using cantilevers coated with 10 μm beads. Mean indentation depth (δ) across all our measurements with its respective standard deviation is also shown. (**c**) Image of AFM cantilever position relative to the neural crest nuclei (left) and nuclei particle image velocity (PIV) displacement heat map result (right) (scale bar 50 μm). c, representative examples from at least 3 independent experiments. Source data

The NC forms at the border of the neural plate^10^ and it is clear that their CCM is mechanically triggered by stiffening of the head mesoderm, a tissue that cells within the NC cluster use as a migratory substrate in vivo^4,11,12^ (Fig. 1a). However, whether the NC adjusts its elastic properties in response to mesoderm stiffening and the molecular mechanism mediating this response remain unknown. To address this, we first measured the apparent elastic moduli (referred to here as stiffness) of wild-type mesoderm and NC from non-migratory to migratory stages by using in vivo atomic force microscopy (iAFM) (Fig. 1b, iAFM controls in Extended Data Fig. 1 and Methods). Our iAFM measurements revealed that NC stiffness is reduced at the onset of CCM, reaching similar values to those registered in the mesoderm at this stage (Fig. 1c). These results indicate that, as proposed in silico and in vitro^8,9^, migrating clusters resting on a soft substrate are not necessarily soft and that stiff surfaces do not always induce cell cluster stiffening. In contrast, we observed that mesoderm stiffening seems to reduce the elastic properties of the NC in vivo.

**Extended Data Fig. 2:**
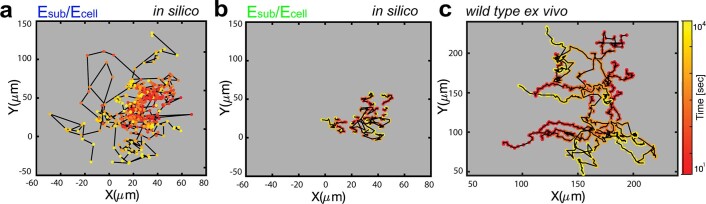
*in silico* and *ex vivo* cell tracks. (**a-b**) Cell tracks extracted from *in silico* simulation under different regimes of E_sub_/E_cell_; (**a**) E_sub_/E_cell_ ~ 1, (**b**) E_sub_/E_cell_ ~ 0.3. (**c**) Cell tracks extracted from *ex vivo* experiments; wild type cells were spreading in a permissive substrate with estimated E_sub_/E_cell_ > 1. All images are representative examples from at least 3 independent experiments or simulations.

In light of these observations, we next sought to gain further insights into the effect of NC stiffness on cell migration by integrating our in vivo AFM data into a three-dimensional active particle computational model using the agent-based framework^13–15^ (Supplementary Note). In the model, individual cells are represented as spherical agents that interact with both other cells within the cluster and with their substrate (inset Fig. 1d). Our simulations take as an input the ratio of the substrate (sub) to cell (i) stiffness (*E*_sub_*/E*_i_) with *E*_i_ in the range of stiffness values recorded in the NC from non-migratory to migratory stages (roughly 150 to 500 Pa), and *E*_sub_ was set at roughly 150 Pa, which is the stiffness of the mesoderm at migratory stages (simulation details in Supplementary Note). Then, the effect of (*E*_sub_*/E*_i_) in the spreading of cells within a cluster was determined through the collective variable radius of gyration squared^16–18^ (*R*_g_^2^, details in Methods). Briefly, the ability of cells within a cluster to directionally spread can be described as an increase in *R*_g_^2^ as a function of time (*t*), $R_{\mathrm{g}}^2\approx t^\gamma$ (inset Fig. 1e). Thus, while low *R*_g_^2^ values with *γ* < 1 as well as short and non-persistent cell tracks report poor migration, larger increases in *R*_g_^2^ values with *γ* > 1 and longer as well as persistent tracks will account for effective and directional migration^17,18^. Our simulations reported that clusters effectively spread at higher *E*_sub_/*E*_i_ values, as shown by rapid increases in *R*_g_^2^ (Fig. 1d). We also observed a high degree of directional motion as revealed by extremely high *γ* values (solid blue line Fig. 1e) and long as well as persistent cell tracks (Extended Data Fig. 2). Consistently, the dynamics of wild-type NC cells spreading from clusters plated on permissive substrates ex vivo, in which an *E*_sub_/*E*_i_ of roughly 1 fitted our in silico observations in terms of *R*_g_^2^ increases and directionality (grey and black lines in Fig. 1e), as well as in cell trajectories (Extended Data Fig. 2). On the other hand, we found that at low values of *E*_sub_*/E*_i_ there is no major change in *R*_g_^2^ (Fig. 1d) and individual cell tracks were short and no persistent reflecting poor and non-directional motion (Extended Data Fig. 2). Our in silico data postulate that if cells within a NC cluster are stiffer than their substrate, they should fail to migrate and that to collectively migrate the NC require reducing their stiffness. This agrees with our measurements at non-migrating stages, where *E*_sub_*/E*_i_ was low (*E*_sub_*/E*_i_ roughly 0.22) when compared to higher *E*_sub_*/E*_i_ recorded at the onset of CCM (*E*_sub_*/E*_i_ roughly 0.90) (Fig. 1c).

## Microtubule deacetylation triggers CCM in vivo

**Fig. 2:**
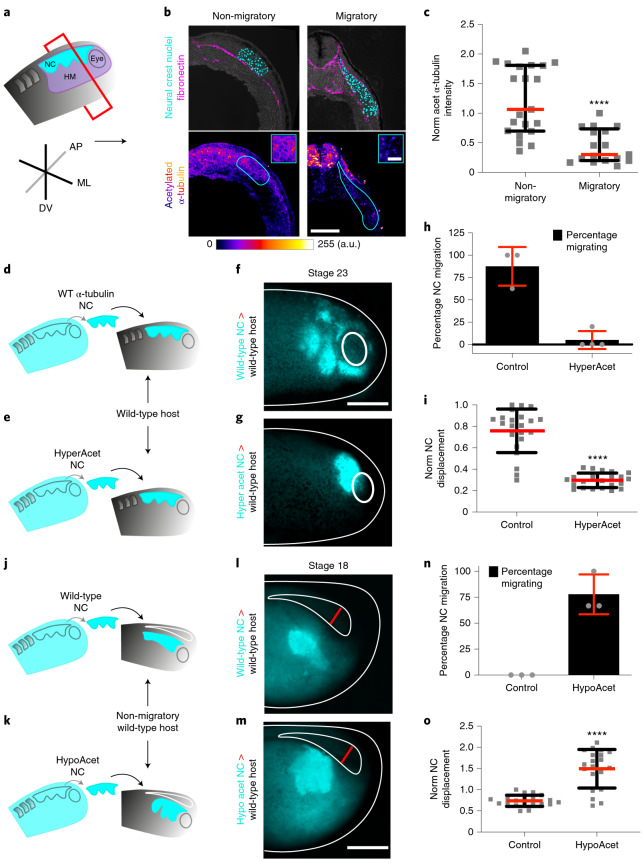
Microtubule deacetylation allows the onset of CCM in vivo. **a**–**c**, NC undergo deacetylation in vivo. **a**, Schematic showing the plane of sectioning (HM, head mesoderm; ML, mediolateral; AP, anteroposterior; DV, dorso-ventral). **b**, In the upper panel, representative confocal projections of transverse cryosections showing highlighted NC nuclei (cyan) and fibronectin (magenta) at non-migratory and migratory stages; in the lower panel, colour-coded projections of the acetylated α-Tubulin channel are shown (scale bar, 100 μm); an inset from the NC region emphasizing the signal differences between both stages is shown in the upper right corner (scale bar, 50 μm). a.u., arbitrary units. **c**, Normalized acetylated α-Tubulin fluorescence intensity; spread of data from the indicated conditions is shown, red lines represent median and whiskers represent interquartile ranges (two-tailed Mann–Whitney ****P < 0.0001, CI = 95%, *n*_non-migratory_ = 17, *n*_migratory_ = 17 embryos). **d**–**o**, Graft experiments. **d**, Diagram of wild-type (WT) stage 17.5 (premigratory) NC grafted into stage 17.5 wild-type host embryos. **e**, Diagram of hyperacetylated stage 17.5 NC grafted into stage 17.5 wild-type host embryos. **f**,**g**, Embryos displaying the results of the grafts shown in **d** and **e**, respectively. **h**, Percentage of embryos displaying NC migration; histograms represent the mean and error bars the s.d. **i**, Normalized displacement of NC along the dorso-ventral axis; red lines represent mean and whiskers s.d. (two-tailed *t*-test, ****P < 0.0001, CI = 95. In **f** and **g**, *n*_control_ = 22, *n*_hyperacetylated_ = 22 animals). **j**, Diagram of wild-type stage 17.5 NC grafted into wild-type stage 14 (non-migratory) host embryos. **k**, Diagram of hypoacetylated stage 17.5 NC grafted into stage 14 wild-type host embryos. **l**,**m**, Embryo displaying the results of the grafts shown in **j** and **k**, respectively. Open neural plate highlighted in white lines with its width indicated by red lines. **n**, Percentage of embryos displaying NC premature migration; histograms represent media, error bars s.d. **o**, Normalized displacement of NC along the dorso-ventral axis; red lines represent mean and whiskers s.d. (two-tailed *t*-test, ****P < 0.0001, CI = 95. In **l** and **m**, *n*_control_ = 20, *n*_hypoacetylated_ = 20 animals). Panels in **b**,**f**,**g**,**l**,**m** are representative examples of at least three independent experiments (scale bars in **f**,**g**,**l**,**m**, 200 μm). Source data

**Extended Data Fig. 3:**
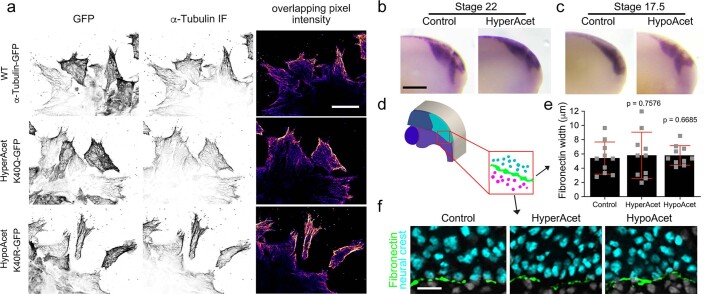
α-Tubulin constructs controls. (**a**) Representative confocal projections showing the signal of wild type α-Tubulin-GFP and mutant α-Tubulin-GFP fused constructs with the signal of α-Tubulin, conditions as indicated. Note that the colocalization of α-Tubulin (IF, immunofluorescence) with the GFP signal of each construct (GFP) was extracted by using the ‘AND’ function of the ImageJ image calculator plugin. Then, colocalizing pixels were color-coded and presented in the right panel column as ‘overlapping pixel signal’ (scale bar 20 μm). (**b, c**) *In situ* hybridisation analysis of NC CCM *in vivo*, lateral views of *sox8* hybridized embryos, treatments as indicated (scale bar 200 μm). Images are representative examples of three independent experiments (note that hyperacetylation blocks migration *in vivo* at stage 22 and hypoacetylation promotes premature migration already at stage 17.5). (**d–e**) Analysis of the impact of NC microtubule acetylation treatments on fibronectin thickness. (**d**) Diagram showing the regions measured to analyze NC grafts (cyan) impact on fibronectin (green) thickness at the interface with the head mesoderm (magenta). (**e**) Chart showing that the layer of Fibronectin has similar thickness in every treatment; bars represent the mean, and whiskers SD (two-tailed t-test, CI = 95%, n = 9 embryos); (**f**) representative confocal projections showing Fibronectin (green) between NC (cyan) and mesoderm (grey) (scale bar, 30 μm). **a**,**b**,**c**,**f**, representative images from at least 3 experiments. Source data

Next, we explored the mechanism by which the NC adjust its elastic properties to collectively migrate, as this would allow us to further validate our model predictions and to experimentally demonstrate the impact of the recorded decrease of NC stiffness in CCM. While several cytoskeletal components contribute to cell stiffness^19^, recent in vitro evidence proposes a central role for microtubule acetylation in tuning cell mechanics both directly and indirectly^20–22^. Given that acetylation of the lysin 40 of α-tubulin (K40-Ac) is relevant for cell motility in vitro^23^, an interesting possibility is that this post-translational modification could mediate the adjustment of NC mechanics in response to mesoderm stiffening. Our in vivo analyses revealed that at non-migratory stages the NC display high levels of microtubule acetylation with subsequent reduction when transiting into migratory stages (Fig. 2a–c). To confirm that this reduction in acetylation is required for the onset of CCM in vivo we grafted control NC expressing wild-type α-Tubulin-GFP or hyperacetylated NC expressing an α-Tubulin mutant that mimics hyperacetylation (K40Q-GFP, ref. ^24^) into wild-type host embryos (Fig. 2d,e). While control NC grafted into wild-type host embryos collectively migrated, hyperacetylated NC displayed inhibition of CCM, as reflected by comparing their net displacement to the control (Fig. 2f–i, controls in Extended Data Fig. 3). Complementarily, we addressed whether the observed reduction in microtubule acetylation (Fig. 2a–c) is sufficient to trigger NC CCM. For this we grafted control or hypoacetylated NC (expressing K40R-GFP, ref. ^24^) into non-migratory wild-type host embryos (Fig. 2j,k). While control NC grafted into wild-type host embryos did not migrate, hypoacetylated NC displayed premature CCM (Fig. 2l–o; controls in Extended Data Fig. 3). Together, these in vivo experiments show that a reduction in microtubule acetylation is essential to allow the onset of NC CCM in vivo.

## Microtubule acetylation modulates cell and cluster stiffness

**Fig. 3:**
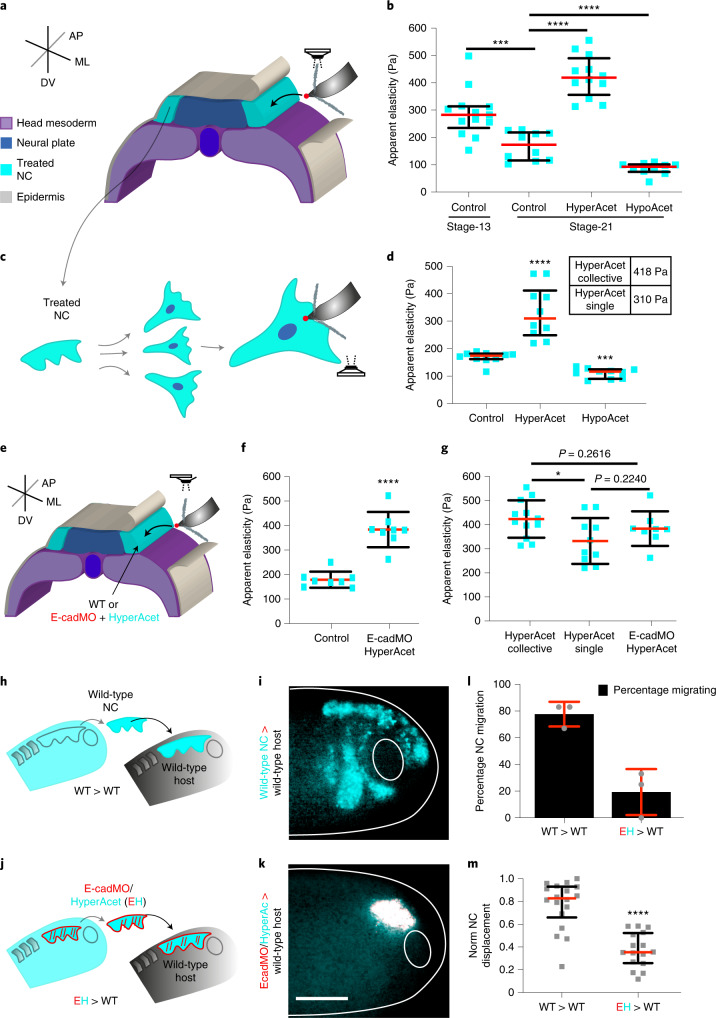
Microtubule deacetylation modulates NC cell stiffness. **a**–**g**, AFM measurements. **a**, Diagrams showing in vivo AFM measurements (ML, mediolateral; AP, anteroposterior; DV, dorso-ventral). **b**, Spread of data for each condition; red lines represent median and whiskers represent interquartile ranges (two-tailed Mann–Whitney test, ****P < 0.0001, ***P = 0.0009, CI = 95%, *n*_ControlNCSt13_ = 13, *n*_ControlNCSt21_ = 10, *n*_HyperacetylatedNCSt21_ = 12; *n*_HypoacetylatedNCSt21_ = 10 embryos; 64 indentations per embryo). **c**, Diagrams showing ex vivo single cell AFM measurements. **d**, Spread of data is shown; red lines represent median and whiskers represent interquartile range (two-tailed Mann–Whitney test, ****P < 0.0001, ***P = 0.0002, CI = 95%, *n*_Control_ = 10, *n*_HyperAcet_ = 10, *n*_HypoAcet_ = 10 cells; 25 indentations per cell). Table inset in **d** compares the stiffness of hyperacetylated NC and clusters obtained in **b** and **d**. **e**, Diagram showing in vivo AFM measurements in control and E-cadherin knockdown (E-cadMO) embryos. **f**, Spread of data is displayed; red lines represent mean and whiskers s.d. (two-tailed *t*-test, ****P < 0.0001, CI = 95%, *n*_Control_ = 8, *n*_EcadMO HyperAcet_ = 8 embryos; 64 indentations per embryo). **g**, Graph compares data from **b**, **d** and **f**; red lines represent mean and whiskers s.d. (two-tailed *t*-test, *P = 0.0221, CI = 95%, *n*_HyperAcetCollective_ = 12 embryos, *n*_HypeAcetSingle_ = 10 cells; *n*_EcadMO+HyperAcet_ = 8 embryos). **h**–**m**, Graft experiments. **h**, Wild-type stage 17.5 (premigratory) NC grafted into wild-type host embryos. **i**, Embryos at stage 24 (migratory) displaying migration. **j**, Stage 17.5 NC from hyperacetylated embryos treated with E-cadherin morpholino were grafted into stage 17.5 wild-type host embryos. **k**, Embryos at stage 24 (migratory) in which migration was inhibited. **l**, Percentage of embryos displaying NC migration; histograms represent mean, error bars s.d. **m**, Normalized displacement of NC along the dorso-ventral axis; red lines represent mean and whiskers represent interquartile ranges (two-tailed Mann–Whitney test, ****P < 0.0001, CI = 95. In **i** and **k**, *n*_WT>WT_ = 18, *n*_EH>WT_ = 16 animals). Panels in **i**,**k** are representative examples of at least three independent experiments (scale bar, 200 μm). Source data

**Extended Data Fig. 4:**
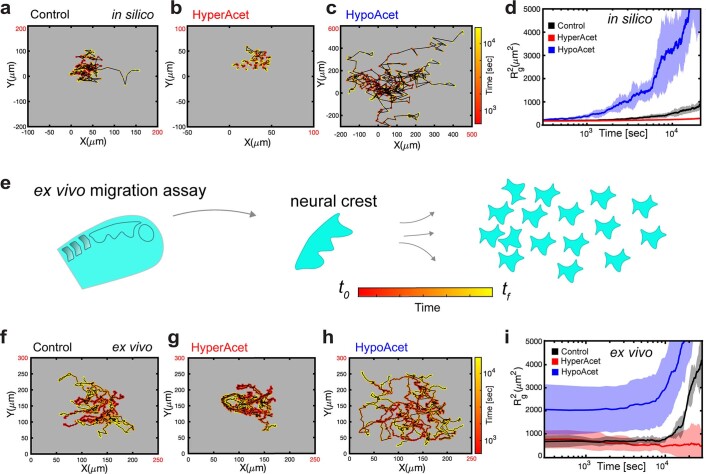
Microtubule acetylation fine tunes cell stiffness to control cell and in turn cluster migration. (**a**–**d**) *In-silico* results for the predicted behaviour of controls, hyperacetylated and hypoacetylated cells and clusters as simulated on stiff substrates. (**a**–**c**) Cell tracks depicting individual cell trajectories (note the differences in the x and y-axes scales when comparing, highlighted in red); (**d**) *R*_*g*_^*2*^ *in silico* calculations showing cell migration under the indicated conditions, line represents mean, and shadow SD. (**e**) Schematic depicts our dispersion assay (detailed in Methods). (**f**–**i**) *ex vivo* results for the behaviour of control, hyperacetylated and hypoacetylated neural crest cells and clusters migrating in a stiff substrate, conditions as indicated. (**f**–**h**) Cell tracks depicting individual cell trajectories; (**i**) *R*_*g*_^*2*^ *ex vivo* calculations showing cell migration under the indicated conditions, line represents mean, and shadow SD. Results displayed here are representative examples from at least 3 independent experiments or simulations. f–i are related to Supplementary Movie 1.

To quantify the impact of microtubule acetylation on NC stiffness we used in vivo AFM (Fig. 3a). As previously observed the stiffness of wild-type NC showed a consistent reduction from non-migratory to migratory stages, but this trend was no longer observed in hyperacetylated NC, as this treatment yielded higher stiffness values (Fig. 3b). On the other hand, hypoacetylated NC displayed low stiffness values when compared to the control (Fig. 3b). Thus, to confirm whether the impact of microtubule acetylation in NC migration can be explained by its influence on NC stiffness, we integrated our AFM results into our theoretical framework. For this, we simulated the behaviour of clusters containing cells with stiffness values recorded from control, hyperacetylated and hypoacetylated NC when migrating in a permissive substrate (Supplementary Note). Our simulations confirmed that while control tracks and *R*_g_^2^ index were consistent with a migratory and directional behaviour, hyperacetylation treatments yielded shorter cell tracks with lower and constant *R*_g_^2^ index, reflecting a poor migratory behaviour of cells within these clusters (Extended Data Fig. 4). On the other hand, hypoacetylation generated rapid and overall higher increases in *R*_g_^2^ that was consistent with large and more directional individual tracks, indicating that low levels of acetylation favour migration (Extended Data Fig. 4). To validate our model predictions more accurately, we analysed the impact of microtubule acetylation in the migratory behaviour of NC clusters containing control, hyperacetylated and hypoacetylated cells in an ex vivo migration assay and extracted experimental *R*_g_^2^ index, and cell tracks for these conditions (Methods and Extended Data Fig. 4). Our ex vivo results reproduced the model predictions with cell migration being reduced by microtubule hyperacetylation and enhanced by hypoacetylation (Extended Data Fig. 4 and Supplementary Video 1). Together, these results indicate that microtubule acetylation affect NC stiffness and with that the onset of CCM.

**Extended Data Fig. 5:**
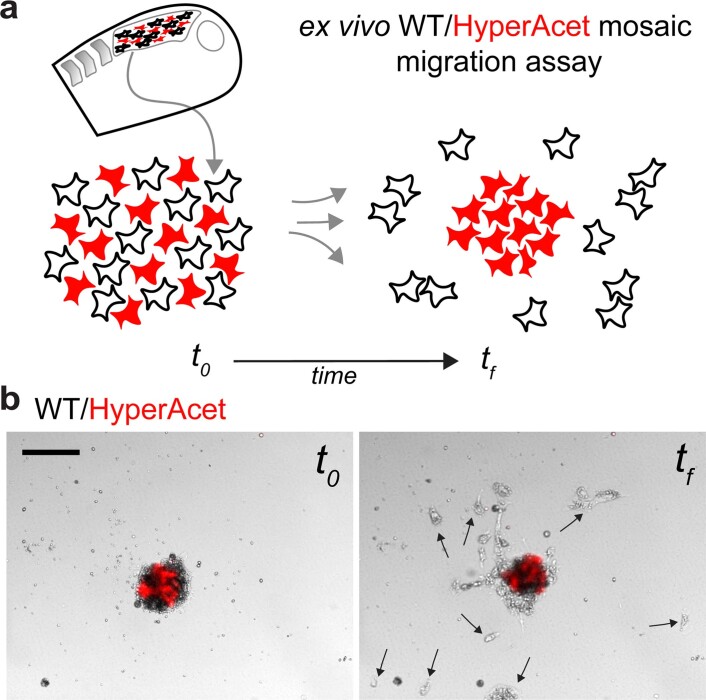
Effects of microtubule acetylation in NC migration are cell autonomous. (**a**) Schematic depicts the dispersion assay of mosaic explants composed of control and Hyperacetylated NC cells. (**b**) Two time points, t_0_ = 0 hours; t_f_ = 11 hours showing the migratory behaviour of a mosaic neural crest cluster. (**b**) representative example from at least 3 independent experiments (scale bar 120 μm). Related to Supplementary Video 2.

**Extended Data Fig. 6:**
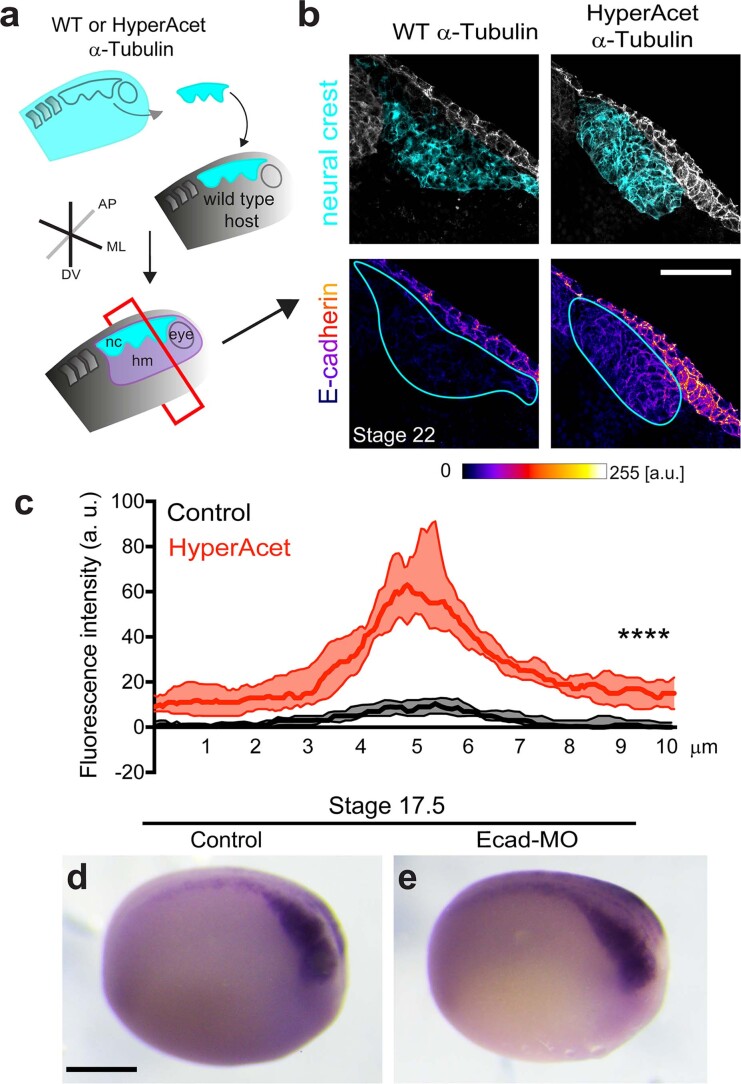
Microtubule acetylation retains E-cadherin at the NC cell-cell contacts. (**a**) Schematic showing a graft experiment and the plane of sectioning post graft (hm, head mesoderm; ML, mediolateral; AP, anteroposterior; DV, dorso-ventral). (**b**) In the upper panel, representative confocal projections of transverse cryosections showing NC from embryos grafted with WT α-tubulin-GFP or HyperAcet α-tubulin-GFP; in the lower panel, colour-coded confocal projections showing the intensity of E-cadherin; the cyan circle delimits the neural crest position (scale bar, 100 μm). (**c**) Chart showing the quantification of E-cadherin max intensity across NC membranes. Fluorescent intensity over a 10 μm distance is shown, middle solid lines represent median and shadow the interquartile range; (two tailed Mann-Whitney test, **** P < 0.0001, CI = 95%, n_control_ = 21, n_HyperAcet_ = 21 junctions). (**e**) Lateral views of embryos hybridised with a probe against *sox8*, conditions as indicated (scale bar 200 μm). b,d,e, representative examples from at least 3 independent experiments. Source data

Next, we sought to gain further insights about the level and the mechanism by which microtubule deacetylation control the observed reduction of NC stiffness. Individual cell tracks extracted from *R*_g_^2^ calculations (Extended Data Fig. 4) and ex vivo mosaic experiments (Extended Data Fig. 5 and Supplementary Video 2) elicited a cell autonomous effect of microtubule acetylation in NC spreading. Thus, we next addressed whether the effects in NC stiffness observed after perturbing microtubule acetylation find their origin at the single cell level. Single cell AFM measurements (Fig. 3c and Methods), confirmed that while hypoacetylation reduces the elastic properties of isolated NC cells, hyperacetylation increases NC cell stiffness (Fig. 3d), reproducing the trend observed when measuring NC clusters in vivo (Fig. 3b). The impact of hyperacetylation in cluster stiffness was slightly higher than its impact at the single cell level (inset table in Fig. 3d). One potential explanation for this could be a retention of E-cadherin at cell–cell junctions^25^; indeed, we tested this and observed that hyperacetylation retained E-cadherin at the NC junctions (Extended Data Fig. 6). Yet, the inhibition of E-cadherin in hyperacetylated NC reveals a partial but not statistically significant effect on the stiffness of NC clusters in vivo (Fig. 3e–g). Furthermore, the inhibition of E-cadherin in hyperacetylated NC did not rescue CCM (Fig. 3h–m) and E-cadherin knockdown per se was not sufficient to promote premature NC migration (Extended Data Fig. 6), unlike what we observed upon microtubule hypoacetylation (Fig. 2j–o). This suggests that while being a hallmark for the mechanically triggered onset of NC CCM^4^, E-cadherin reduction may not be the main contributor to the decrease of NC stiffness. Altogether, these results indicate that microtubule deacetylation is one of the main components of the mechanism that reduces NC cell and in turn cluster stiffness to allow the onset of CCM in vivo.

## Substrate stiffening promotes microtubule deacetylation

**Fig. 4:**
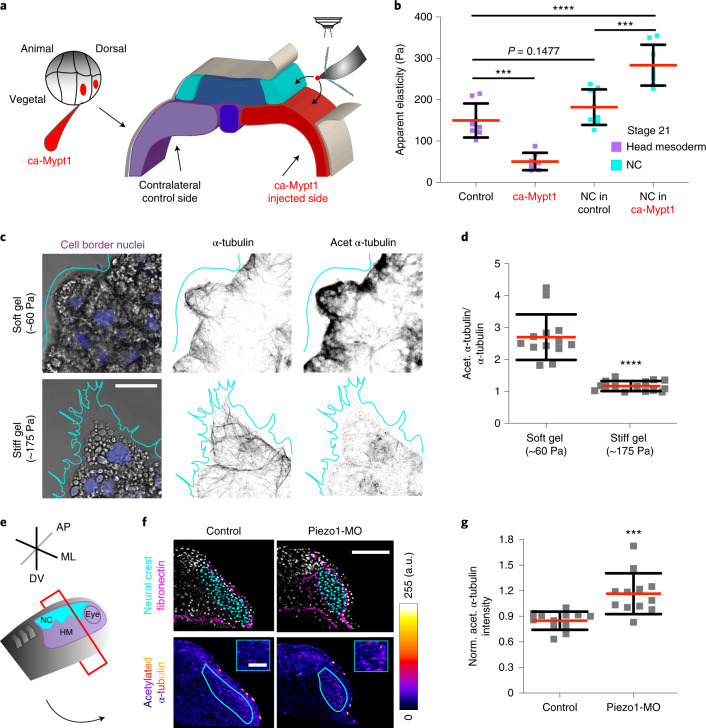
Mesoderm stiffening control microtubule deacetylation via Piezo1-mediated mechanosensing. **a**, Schematic showing the regions measured by AFM, black arrows indicate the recorded region. **b**, Spread of data for each condition; red lines represent mean and whiskers s.d. (two-tailed *t*-test ****P < 0.0001, ***P < 0.0006, CI = 95%, *n*_control mesoderm_ = 8, *n*_ca-Mypt1 mesoderm_ = 7, *n*_migratory NC in control_ = 8, *n*_migratory NC in ca-Mypt1_ = 8 embryos; 64 indentations per embryo). **c**, Immunofluorescence analysis showing acetylated α-Tubulin and α-Tubulin signal in NC platted on soft or stiff hydrogels (nuclei in magenta and NC border in cyan) (scale bar, 20 μm). **d**, Normalized fluorescence intensity ratio of acetylated α-Tubulin versus α-Tubulin; red lines represent mean and whiskers s.d. (two-tailed *t*-test, ****P < 0.0001, *n*_soft_ = 13 clusters; *n*_stiff_ = 15 clusters). **e**–**g**, Piezo1 regulates microtubule acetylation in vivo. **e**, Schematic showing the plane of sectioning (HM, head mesoderm; ML, mediolateral; AP, anteroposterior; DV, dorso-ventral). **f**, Upper panel, confocal projections of transverse cryosections showing highlighted NC nuclei (cyan) and fibronectin (magenta); lower panel, colour-code image of the acetylated α-Tubulin channel (scale bar, 100 μm); upper right inset emphasizes the signal differences between both treatments in the NC (scale bar, 50 μm). **g**, Normalized acetylated (norm. acet.) α-Tubulin fluorescence intensity, spread of data is shown; red lines represent mean and whiskers s.d. (two-tailed *t*-test ***P = 0.004, CI = 95%, *n*_Control_ = 12, *n*_Piezo1-MO_ = 12 embryos). **c** and **f** are representative confocal projections from three independent experiments. Source data

**Extended Data Fig. 7:**
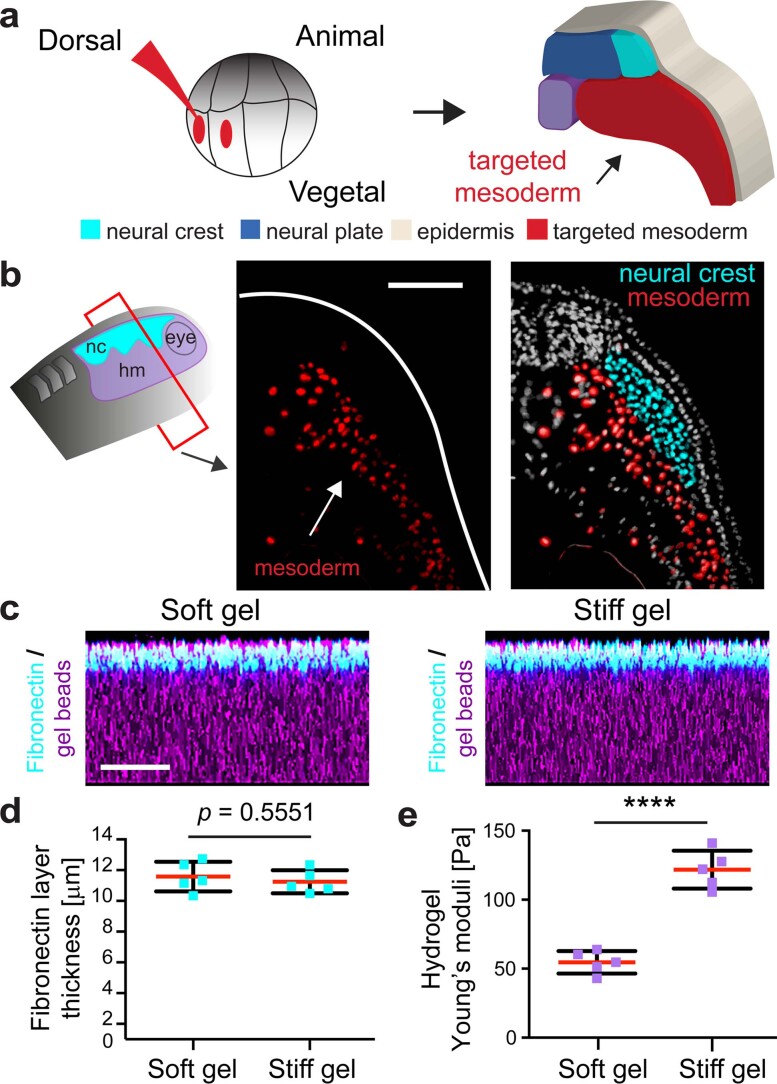
Targeted injections into the head mesoderm and hydrogels system validations. (**a**) Schematic displaying targeted injections into the mesoderm. (**b**) Drawing shows the plane of cryosection (hm, head mesoderm), and confocal projections display the result of targeting nuclear RFP into the head mesoderm (red); NC nuclei are also shown (cyan) (scale bar, 150 μm). (**c**–**e**) Characterisation of the *ex vivo* system that reproduces the stiffness values that neural crest cells experience at non- and migratory stages. (**c**) Orthogonal view of a confocal projection of soft and stiff hydrogels. Images in (**c**) are representative examples from at least 3 independent experiments (scale bar, 50 μm). (**d**) Chart showing that the layer of Fibronectin has similar thickness in soft and stiff gels; spread of data is shown; data points represent the mean obtained from each gel and at least 5 measurements were taken per gel; red line represents mean, and whiskers show SD (two-tailed t-test ****P < 0.0001, CI = 95%, n = 5 gels;). (**e**) AFM measurements obtained from soft and stiff hydrogels; spread of data is shown; each data point represents the mean of a gel, and 64 indentations were performed per gel; red lines show mean and whiskers SD; n = 5 gels; two-tailed t-test, ****P < 0.0001, CI = 95% (scale bar, 50 μm). **b**, representative examples from at least 3 independent experiments. Source data

**Extended Data Fig. 8:**
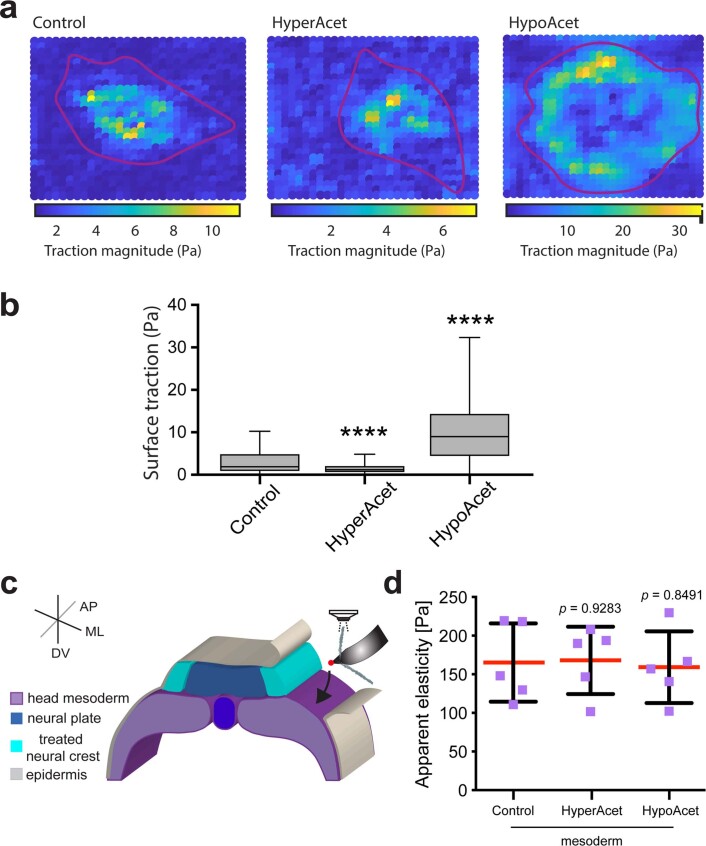
Impact of NC microtubule acetylation in traction force and mesoderm stiffness. (**a**) Heat map showing traction force microscopy results (detailed in Methods); conditions as indicated in the figure. (**b**) Box plot showing the traction force exerted by NC clusters in Pascals (Pa), box represents the interquartile range, central line represent median, and error bars the min and max values (two-tailed Mann-Whitney test, ****P < 0.001, CI = 95%; n = 9 clusters). (**c**) Schematic showing the *i*AFM measurements (black arrow points the mesoderm) in wild-type or treated embryos, note that in this experiment we only measured mesoderm after modifying NC acetylation as indicated. (**d**) Chart showing spread of data, red lines represent mean, whiskers SD; two-tailed t-test, CI = 95%, n _control_ = 5, n _HyperAcet_ = 5, n _HypoAcet_ = 5 embryos; 64 indentation were performed per embryo). Source data

Next, we asked whether mesoderm stiffening, which is known to trigger NC migration^4^, mediates the decrease of NC stiffness by fine-tuning microtubule acetylation. To test this, we first softened the mesoderm by using a method relying on the targeted injection of an active form of myosin phosphatase-1 (ca-Mypt1)^4,26^ (Fig. 4a, Extended Data Fig. 7 and Methods). Targeted injection of ca-Mypt1 was sufficient to decrease mesoderm stiffness, as we have previously shown^4^ and to inhibit the decrease in NC stiffness, by maintaining similar levels to those observed in wild-type non-migratory embryos (Fig. 4b). Then, to corroborate whether microtubule deacetylation also depends on mesoderm stiffening we used a controlled ex vivo environment that mimics the stiffness cells within NC clusters experience at non-migratory and migratory stages^4^ (Methods and Extended Data Fig. 7). In agreement with our in vivo observations (Fig. 2b,c), cells within NC clusters plated on soft substrates displayed high levels of microtubule acetylation but these levels were drastically reduced in cells from clusters plated on stiff surfaces (Fig. 4c,d). Moreover, from our model we can extract that modifying cell stiffness should feedback into the interaction of cells with their substrate via force generation, which is required for cell movement and comparable to experimental cell traction force (Supplementary Note). As a consequence, our traction force microscopy (TFM) analyses showed that microtubule acetylation affect the traction of NC clusters ex vivo (Extended Data Fig. 8). Notably, modifying microtubule acetylation in vivo did not alter mesoderm stiffness (Extended Data Fig. 8).

## Piezo1 mechanosensing mediates microtubule deacetylation and CCM

**Extended Data Fig. 9:**
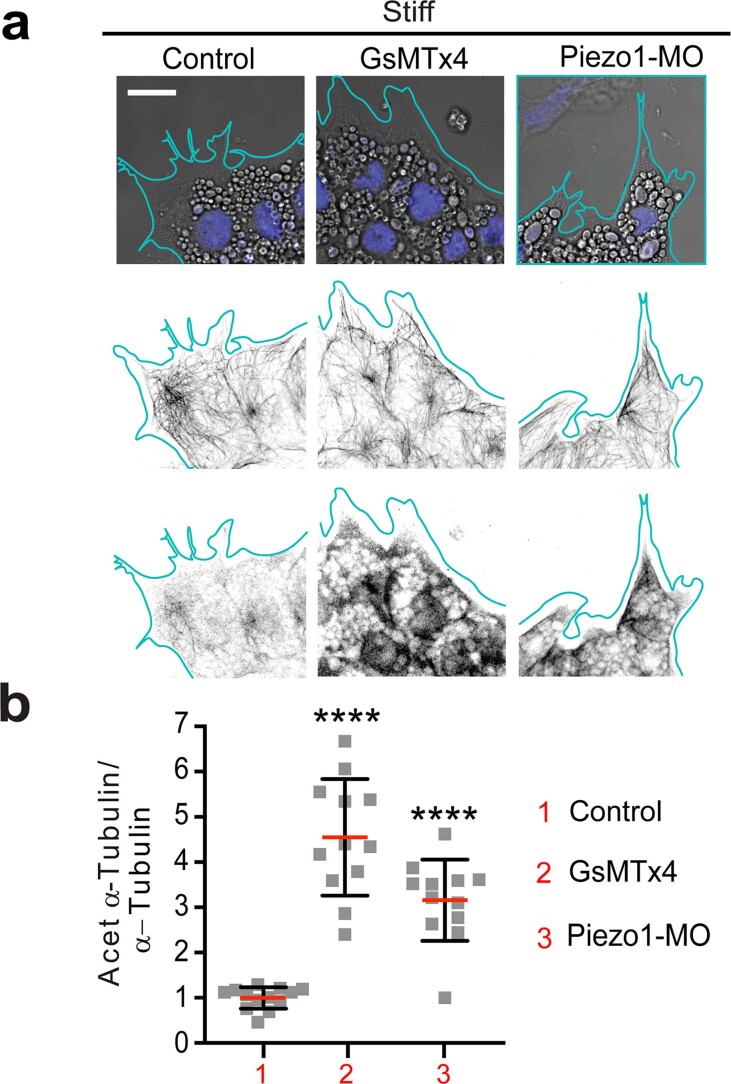
Piezo1 mechanosensing mediates NC microtubule acetylation. (**a**) Immunofluorescence analysis showing acetylated α-Tubulin and α-Tubulin signal in NC plated in permissive substrates (nuclei is shown in magenta and NC border is depicted by cyan solid lines); treatments and channels as indicated. Images are representative examples from at least 3 independent experiments (scale bar 25μm). (**b**) Chart showing the normalised fluorescence intensity ratio of acetylated α-Tubulin vs α-Tubulin; spread of data is shown, red lines represent mean, whiskers represent SD (two tailed t-test ****P < 0.0001, CI = 95%, n_1–3_ = 37 cells). a, representative examples from at least 3 independent experiments. Source data

We next explored the molecular mechanism by which NC sense and translate mesoderm stiffening into deacetylation. To shed light on this, we inhibited membrane mechanosensing by performing incubations with GsMTx4, an inhibitor of stretch activated channels (SACs)^27^. GsMTx4 incubation led to high levels of acetylation when comparing treated and control NC clusters (Extended Data Fig. 9). But as GsMTx4 inhibits several SACs, we next searched for specific SACs that could mediate this effect in the NC. RNA-sequencing (RNA-seq) libraries from isolated migratory NC (Methods) revealed that the stretch activated channel Piezo1—a well-established mechanosensor^28,29^—is expressed by NC cells (Supplementary Table 1). Thus, we tested the role of Piezo1 on microtubule acetylation by using a validated morpholino designed to knockdown *Xenopus* Piezo1 (Piezo1-MO)^30,31^. Piezo1 knockdown in the NC led to increased microtubule acetylation levels both ex vivo (Extended Data Fig. 9) and in vivo (Fig. 4e–g), revealing a role for Piezo1 in mediating microtubule acetylation in native contexts.

**Fig. 5:**
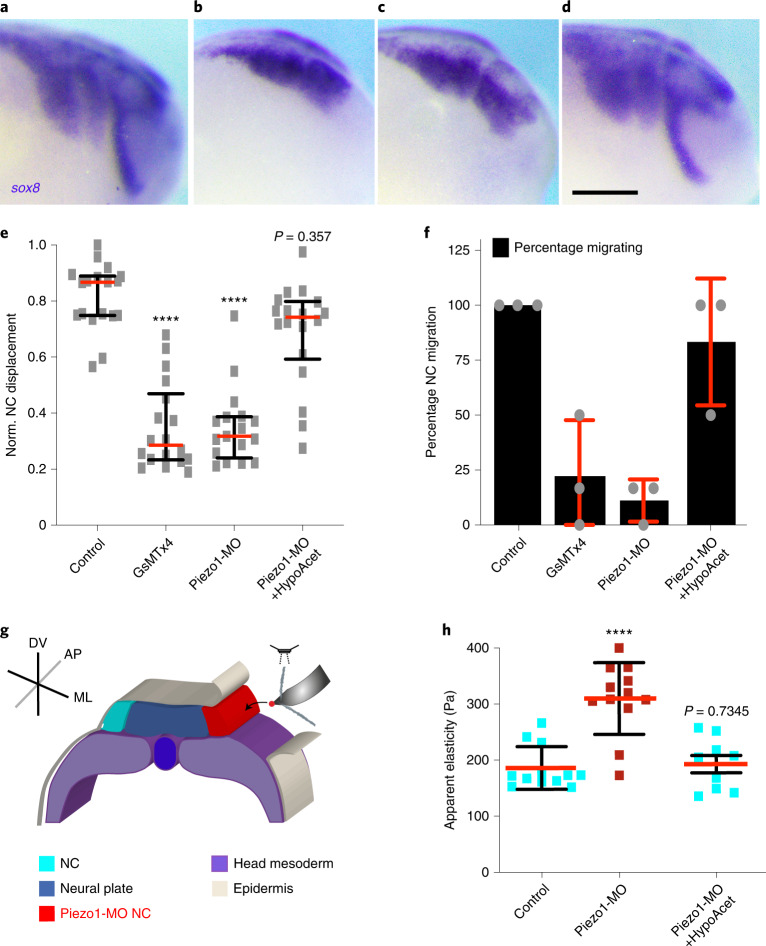
Piezo1 fine-tunes NC mechanics via microtubule acetylation to allow the onset of CCM in vivo. **a**–**f**, In situ hybridization analysis of NC CCM in vivo. **a**–**d**, Lateral views of *sox8* hybridized embryos (scale bar 200 μm): treatments; control (**a**), GsMTx4 (**b**), Piezo1-MO (**c**) and Piezo1-MO+HypoAcet (**d**). **e**, Normalized (norm.) displacement of NC along the dorso-ventral axis; red lines show median and whiskers represent interquartile ranges (Kruskal–Wallis test *P* < 0.0001, CI = 95%, *n*_control_ = 17, *n*_GSMx4_ = 18, *n*_cPiezo1-MO_ = 17, *n*_rescue_ = 16 embryos). **f**, Percentage of embryos displaying NC migration histograms represent media, error bars are s.d. Panels are representative examples from at least three independent experiments. **g**,**h**, iAFM measurements. **g**, Diagram showing iAFM measurements in control or Piezo1-MO treated NC (ML, mediolateral; AP, anteroposterior; DV, dorso-ventral). **h**, Spread of data for each condition is shown; red lines represent mean and whiskers s.d. (two-tailed *t*-test, ****P < 0.0001, CI = 95%, *n*_Control NC_ = 12, *n*_Piezo1-MO NC_ = 12 embryos, *n*_Piezo1-MO+HypoAcet NC_ = 9 embryos). Source data

**Extended Data Fig. 10:**
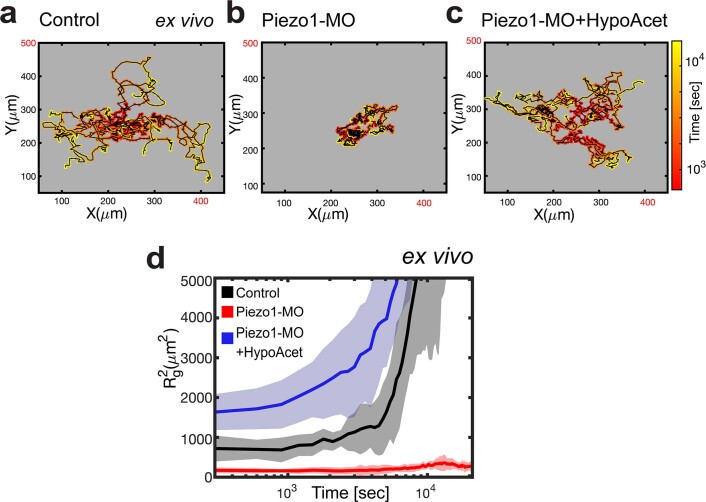
Piezo1 modulate NC cell spreading *ex vivo*. (**a**–**d**) *ex vivo* results for the behaviour of Control, Piezo1-MO, and Piezo1-MO + hypoacetylated NC cells and clusters when migrating on a stiff permissive substrate; conditions as indicated in the figure. (**a**–**c**) Cell tracks depicting individual cell trajectories; (**d**) *R*_*g*_^*2*^ *ex vivo* calculations showing collective spreading under the indicated conditions, line represents mean, and shadow SD. **a**–**c**, representative examples of at least 3 independent experiments. Related to Supplementary Movie 3.

In addition to these effects in microtubule acetylation, both GsMTx4 incubation and the targeted knockdown of Piezo1 in NC drastically impaired NC CCM in vivo (Fig. 5a–c,e,f). Furthermore, our ex vivo analysis of *R*_g_^2^ index revealed that the migratory ability of Piezo1 knockdown cell and clusters were reduced when compared to the control (Extended Data Fig. 10 and Supplementary Video 3). Since Piezo1 controls several cellular processes^32^ one possibility is that the observed effects on cell migration may be due to off-target effects. To address this, we performed an epistatic experiment in which Piezo1-MO was injected into hypoacetylated NC (Piezo1-MO + HypoAcet). This co-injection was sufficient to rescue the effect of Piezo1 knockdown in NC migration in vivo and ex vivo, confirming the specificity of our results (Fig. 5d–f, Extended Data Fig. 10 and Supplementary Video 3). Next, to confirm that the defects in NC microtubule acetylation and CCM observed on Piezo1 knockdown are related to cell stiffness we measured the impact of Piezo1-MO in NC stiffness by using iAFM. Our measurements revealed that Piezo1-MO injection in the NC was sufficient to cell-autonomously abolish the decrease of NC stiffness that we observed at the onset of CCM in wild-type embryos and that hypoacetylation was also sufficient to rescue this effect (Fig. 5g,h). These results indicate that Piezo1 is required to fine-tune NC mechanics in response to mesoderm stiffening by allowing microtubule deacetylation and in turn CCM in vivo.

## Soft clusters migrate in compliant native substrates

**Fig. 6:**
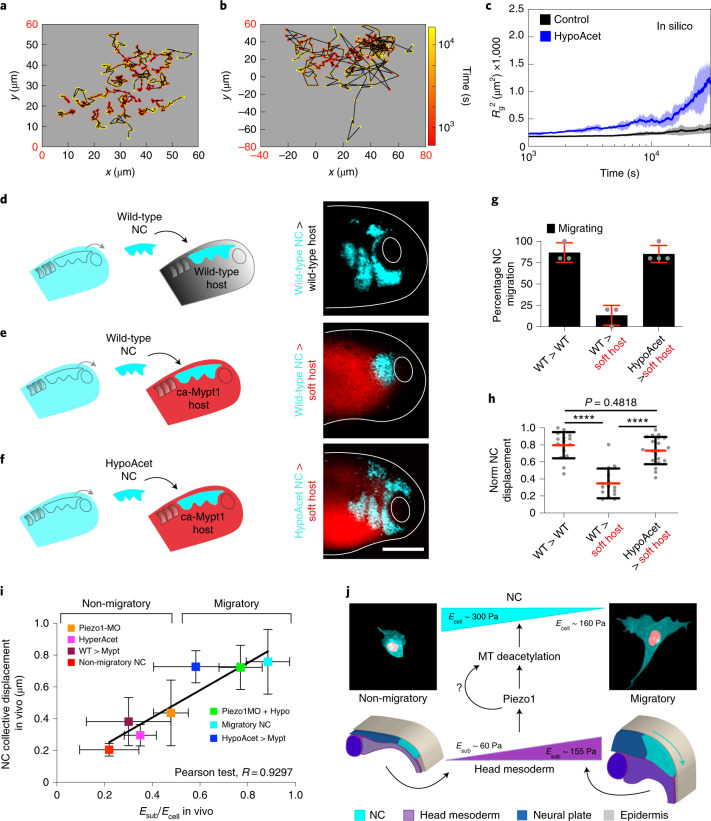
Soft cell clusters migrate in compliant native environments. **a**–**c**, In silico results for the predicted behaviour of controls and hypoacetylated cells and clusters plated on soft substrates. **a**,**b**, Cell tracks depicting individual cell trajectories (note the differences in the *x* and *y* axes scales when comparing, highlighted in red): control (**a**) and HypoAcet (**b**). **c**, In silico *R*_g_^2^ calculations showing collective cell behaviours under the indicated conditions: solid lines represent mean and shade the s.d. **d**–**h**, Graft experiments. **d**, Wild-type premigratory (stage 17.5) NC grafted into wild-type host embryos. **e**, Wild-type premigratory NC grafted into softened host embryos. **f**, Hypoacetylated premigratory NC grafted into softened hosts (scale bar, 200 μm). **g**, Percentage of embryos displaying NC migration; histograms represent mean and error bars s.d. **h**, Normalized displacement of NC along the dorso-ventral axis; red lines represent mean and whiskers s.d. (two-way ANOVA *P* < 0.0001; two-tailed *t*-test, ****P < 0.0001, CI = 95. In **g** and **h**, *n*_wt into wt_ = 16, *n*_wt into softened_ = 15, *n*_hypoacetylated into softened_ = 20 embryos). Scale bar, 200 μm. **d–f** are representative examples from at least three independent experiments. **i**, Summary of the strong correlation we found between the cell-to-substrate stiffness ratio obtained from all our in vivo AFM measurements and the net displacement of NC along the embryo in under the same treatments (Pearson test, *R* = 0.9297). **j**, Schematic providing a mechanistic overview on how the mechano-molecular feedback loop that underly onset of CCM in vivo operates (detailed in Outlook). Source data

Considering our results, our next goal was to dissect whether NC require a threshold value of substrate stiffness to migrate or whether lowering their elastic properties to match softer substrates would be sufficient to allow CCM. Since we found that microtubule deacetylation reduces NC stiffness to allow CCM in vivo, we next tested whether hypoacetylation is sufficient to allow CCM in compliant substrates, in which wild-type cells do not normally migrate^4^. As an initial approach, we simulated migration of cells within clusters at control and hypoacetylated stiffness values when exposed to a soft substrate that resembles the stiffness of a non-migratory mesoderm (roughly 50 Pa). Our simulations confirmed that control clusters struggle to migrate on soft substrates as reflected by the shorter length of their individual tracks and flat *R*_g_^2^ index (Fig. 6a,c). Unlike those, individual cell tracks and *R*_g_^2^ values of hypoacetylated NC clusters elicited a migratory behaviour on these soft substrates (Fig. 6b,c), suggesting that hypoacetylated cells could migrate in compliant surfaces. To confirm these results in vivo, the migration of wild-type or hypoacetylated NC was assessed after grafting into wild-type embryos or into embryos with softened mesoderm. As expected, wild-type control NC clusters grafted into wild-type hosts collectively migrated, but their migration was inhibited when grafted into softened embryos (Fig. 6d,e,g,h). Hypoacetylated NC effectively migrated by following stereotypical paths when grafted into these softened native environments (Fig. 6f–h). As a consequence, with these observations we found a strong correlation between the *E*_sub_*/E*_i_ calculated from our in vivo AFM data and the net distance that NC migrated in embryos under all the treatments we analysed (Fig. 6i). Thus, on the basis of these results it is tempting to speculate that a threshold value of substrate stiffness may not be as essential for CCM as it is achieving an optimal *E*_sub_*/E*_i_ ratio. Yet, our results also show that reducing cell and in turn cluster stiffness requires substrate stiffening (Fig. 4b). Thus, we propose that a ‘stiff substrate’ is not only a permissive platform that supports CCM, but that substrate stiffening play a major informative role in the mechano-molecular feedback loop by which cells within clusters attain an optimal cell-to-substrate stiffness ratio to migrate in mechanically dynamic and often compliant native substrates.

Collectively, our work reveals that substrate stiffening leads to a reduction in the stiffness of cells within migrating clusters and that this unsuspected mechanical cellular response is essential for CCM in vivo, as it allow clusters to achieve an optimal cell-to-substrate stiffness ratio in response to substrate stiffening. Mechanistically, we found that this substrate mediated reduction on NC stiffness is achieved via Piezo1 regulation of microtubule deacetylation (Fig. 6j). Thus, our data have the potential to affect our approach to several physiological and pathological processes that require CCM, such as embryogenesis, tissue repair and cancer invasion.

## Outlook

According to in vitro results, cells within clusters resting on soft substrates are softer than when exposed to a stiffer environment^7,33,34^. Despite this, recent evidence argues that cell and substrate stiffness are independent when cells are plated onto compliant surfaces^8,9^, with particular implications for cancer^11^. Our in vivo work reinforces this idea as we observed that NC cells and, in turn, cluster stiffness are higher than the stiffness recorded in the head mesoderm (its substrate) and that mesoderm stiffening induces NC softening, offering an alternative or complementary scenario to the current view on how cell stiffness is influenced by substrate stiffening. These results are also relevant for our understanding of cancer biology as in some malignant contexts cancer cells became softer^8,35^.

Furthermore, our data reveal that microtubule acetylation affect the stiffness of individual NC cells to control cluster mechanics and migration. Still, whether microtubule acetylation affect cell stiffness in a direct or indirect manner remains elusive. On the basis of the current knowledge in the field we foresee at least three scenarios: (1) that microtubule acetylation could directly affect cell mechanics, as acetylated microtubules are more stable and stiffer, unlike deacetylated microtubules^20,36^; (2) that microtubule acetylation operates by controlling the activity of GEF-H1 and with that actomyosin contractility, as it has been recently proposed in vitro^22,37^ and (3) a combination of scenarios 1 and 2 may emerge owing the complexity and dynamic nature of the in vivo migratory environment. Regardless, whether our observations are due to a direct or indirect effect, our data position microtubule deacetylation as a key player of the mechano-molecular feedback loop by which NC cells mediate the reduction in cluster stiffness at the onset of CCM in vivo.

On the other hand, our data on E-cadherin confirmed that the elastic properties of NC cells and in turn clusters are primarily, but eventually not exclusively, controlled by microtubule acetylation. Still switching E- to N-cadherin is a hallmark of NC epithelial-to-mesenchymal transition^38^, and we have previously shown that substrate stiffening is involved in this cadherin switch^4^. Thus, our current study invites to further dissect the hierarchical or relative contribution of microtubule acetylation and cadherin switches to the onset of CCM in NC and eventually in other cellular contexts.

Moreover, we reveal that Piezo1-mediated mechanosensing controls NC microtubule deacetylation, cell mechanics and CCM in vivo. Still, the molecular signalling by which Piezo1 controls microtubule acetylation and whether this mechanosensitive pathway operates in other biological contexts remains to be determined. Another relevant question is how force that is sensed at the substrate is transmitted across the 3D NC cluster. This topic is under intense research and several studies point to the role of cell–cell junctions as mediators of mechanical force across migrating clusters^39–41^. Indeed, N-cadherin can transmit traction force^37,39^ and the NC requires N-cadherin to collectively migrate^42^. Thus, an interesting possibility is that N-cadherin may transduce force from the mesoderm across the NC cluster during CCM.

We also showed that inducing hypoacetylation was sufficient to allow CCM in soft native substrates. This result confirmed that lowering cell stiffness is sufficient for cells to migrate regardless of the mechanical nature of their environment. Further studying these observations can affect our understanding of processes such as cancer cell migration, as recent data indicate that these cells often migrate across soft viscoelastic native tissues^8,43^, such as the ones reported here. In addition, we can expect that when experiencing a soft non-migratory surface, Piezo1 activity may be low in the NC. This lack of active mechanosensing could eventually explain why cells do not adjust their stiffness to the substrate in these compliant contexts, as they do in stiff substrates, further confirming the instructive nature of substrate stiffening.

Since microtubule acetylation, cell-substrate mechanics and CCM are essential for a variety of biological processes such as embryo development, tissue repair and cancer, we predict that our observations will be of general interest across the biological and physical sciences. Broadly, our data contribute to the growing body of evidence arguing that mechano-molecular feedback loops, such as the one described here, coordinate morphogenesis in physiology and disease^4,44–46^.

## Methods

All animal experiments were approved by Ethics Committee and the Animal Welfare Body of the Instituto Gulbenkian de Ciencia (IGC) and by the Direção Geral de Alimentação e Veterinária. All institutional, project and personal licences are in place.

### *X. laevis* manipulation to obtain embryos

Adult animals were maintained at 18 °C in a temperature-controlled environment and embryos were obtained by in vitro fertilization^38^. Briefly, induction of ovulation was performed in adult females by injecting chorionic gonadotrophin (Intervet); after ovulation, oocytes were in vitro fertilized by mixing with a sperm solution. On successful fertilization, embryos were staged by following developmental tables^47^ and maintained between 14 and 18 °C.

### In situ hybridization and riboprobes and messenger RNA in vitro transcriptions

In situ hybridizations were performed by following a step by step protocol^48^. In brief, an antisense template DNA for the NC marker *sox8* (ref. ^49^) was generated by linearizing with EcoRI (New England Biolabs). Then a digoxigenin-labelled probe against *sox8* (ref. ^49^) was transcribed in vitro by using this linearized plasmid as a template and by following the instructions of a commercial in vitro Transcription System (Promega P1420). Templates for wild-type α-Tubulin-GFP (Addgene 56450); hyperacetylated α-Tubulin (K40Q-eGFP, Addgene 105302) and hypoacetylated α-Tubulin (K40R-eGFP, Addgene 105302) were generated by PCR, using the following primers: T7-promoter containing forward primer 5′-ggaggtctatataagcagagtaatacgactcactataggctggtttagtgaaccgtc-3′ and a reverse primer 5′-tacgcgttaagatacattgatgagtttggacaaaccacaacta-3′. Transcription was performed with a T7 transcription kit (mMESSAGE mMACHINE, AM1334 for T7). Templates for all other mRNA in vitro transcriptions (membrane GFP (mGFP), nuclear RFP (nRFP), CA-MYPT1) were generated by digesting with NotI (New England Biolabs) and transcribed with an Sp6 transcription kit by following the fabricant instructions (mMESSAGE mMACHINE SP6, Thermo-Fisher AM1340 for Sp6).

### Morpholino and mRNA injections

Fertilized eggs were dejellied for 5 min with a solution containing 0.5 g of cysteine (Sigma) and 500 μl of 5 N NaOH, dissolved in 25 ml of ddH_2_O. All injections were performed with pulled glass needles that were calibrated to inject 10 nl on a gas pulse of 20 psi for 0.2 s. Depending on the type of experiment, different stages and/or blastomeres were injected (specified in each figure). For cell labelling, 250 pg of mGFP and or nRFP were injected per blastomere. For targeted NC injections, embryos at eight-cell stage were injected near the division point of a dorsal and a ventral blastomeres of the animal pole, with: 17 ng of a morpholino designed against *Xenopus Piezo1* (Piezo1*-*MO 5′-CACAGAGGACTTGCAGTTCCATCCC-3′); or 22 ng of *Xenopus* E-cadherin (Ecad-MO 5′-AACCAGGGCCTCTTCAACCCCATTG-3′). These morpholinos were synthesized by GeneTools and previously validated in *X. laevis* Piezo1-MO (ref. ^30^) and Ecad-MO^50^. The same strategy was used to inject wild-type α-Tubulin-GFP; hyperacetylated α-Tubulin and hypoacetylated α-Tubulin^24^. For targeted mesoderm injections, 1 ng of CA-MYPT^4^ or mGFP plasmids were injected into dorso-vegetal blastomeres at the 16-cell stage.

### GsMTx4 incubations in vivo and ex vivo

For GsMTx4 incubations, embryos were incubated in a solution containing 100 μM of GsMTx4 (08GSM001, Smartox TebuBio) dissolved in dimethyl sulfoxide (Thermo-Fisher). Embryo incubations were performed from stage 13 (non-migratory) to stage 22 (migratory) and immediately processed for in situ hybridization. For ex vivo incubations, NC explants were taken from embryos at stage 15 (non-migratory) (as described below). Then NC clusters were let to attach and spread in a fibronectin dish for 30 min, incubated with 5 μM GsMTx4 for roughly 3 h and immediately processed for immunofluorescence.

### Ex vivo NC culture, spreading assay and graft experiments

#### NC dissection

Devitellinized embryos were placed in a dish containing plasticine and filled with embryo media Marc’s Modified Ringer (containing 0.2 mM CaCl_2_·2H_2_O, 10 mM NaCl, 0.1 mM MgCl_2_·6H_2_O, 0.2 mM KCl, 0.5 mM HEPES with pH 7.1–7.2). Embryos were immobilized by gently holding them with plasticine and the epidermis was removed with a hair-knife tool. The NC was anatomically identified and removed with the hair knife. Explants were transferred to a dish containing Danilchik’s medium (1 mM MgSO_4_(7H_2_O), 5 mM Na_2_CO_3_, 4.5 mM KGluconate, 53 mM NaCl, 32 mM NaGluconate, 0.1% BSA and 1 mM CaCl_2_; pH was adjusted to 8.3 with Bicine).

#### Dispersion assay

To analyse the migration of NC ex vivo we used a collective spreading or dispersion assay. To do this, dissected NC clusters were platted into a fibronectin-coated glass bottom dish (μ-Dish, 35 mm diameter, Ibidi). NC were allowed to attach, and their migration and dispersion was recorded by time-lapse. These assays have been widely used as a readout of the ability of cells within a NC cluster to migrate, mostly because in the absence of constrains or biasing cues wild-type clusters radially spread. This allows for the analysis and comparison of several motility parameters among treatments^48^.

#### Graft experiments

NC explants were removed as described in NC dissection. Then the donor NC was carefully placed into host embryo by using a hair knife. To hold the grafted NC in place, a piece of cover-glass (0.1 mm thick) was positioned over the grafted NC. After roughly 1 h, the coverslip was removed, and the embryos were imaged when reaching the stages required for each experiment.

### Polyacrylamide (PAA) hydrogels preparation

Soft gel mixes contained: 550 µl of 7.6 mM hydrochloric acid (HCL), 330.5 µl of double-distilled water (ddH_2_O), 0.5 µl *N*,*N*,*N*′,*N*′-tetramethylethylenediamine (TEMED) (Sigma), 20 µl 2% *bis*-acrylamide (BioRad), 70 µl of 40% acrylamide (BioRad), 20 µl 0.1 M NHS (*N*-hydroxysuccinimide, Sigma-Aldrich), 4 µl of 200 nm diameter beads resuspended at 0.2 µM (Invitrogen) and 5 µl of 10% ammonium persulfate (GE HealthCare) (prepared just before use). Stiff gels mixes contained: 550 µl of 7.6 mM HCL, 258.5 µl of ddH_2_O, 0.5 µl of TEMED (Sigma), 25 µl 2% *bis*-acrylamide (BioRad), 137 µl of 40% acrylamide (BioRad), 20 µl of 0.1 M NHS (*N*-hydroxysuccinimide, Sigma-Aldrich), 4 µl of 200 nm diameter beads resuspended at 0.2 µM (Invitrogen) and 5 µl of 10% ammonium persulfate (GE HealthCare) (added just before use). A 12-μl drop of PAA mix was placed into the hydrophilic glass of a glass bottom dish (FD5040-100). The PAA mix was covered with a hydrophobic 13-mm diameter × 0.1 mm glass coverslips that were prepared fresh by coating them with PlusONE Repel-Silene ES (GE Healthcare) for 15 min at room temperature and dried with an air pistol. Polymerization proceeded for 45 min at room temperature in a humidifier chamber. The coverslip was carefully removed, and gels were washed three times for 2 min with 10 mM HEPES.

### Gel functionalization

Fibronectin was covalently linked to the soft or stiff gels by immersion of the gels into a solution containing 0.2 M EDC ((1-ethyl-3-(3-dimethylaminopropyl)carbodiimide hydrochloride), Calbiochem), 0.1 M NHS (*N*-hydroxysuccinimide, Sigma-Aldrich), in 0.1 M MES buffer (in milliQ water, pH 5.0, 2-(*N*-morpholino)-ethane sulfonic acid, Sigma-Aldrich). After washing twice with PBS, gels were incubated with 0.1 mg ml^−1^ of fibronectin for 1 h 45 min at room temperature. Fibronectin was washed with PBS and the crosslinking-reaction was quenched by incubating the gels for 15 min with 0.32% ethanolamine (Sigma-Aldrich) in PBS. Fluorescent Fibronectin (Cytoskeleton, Inc., HiLyte 488) was used to determine gel functionalization (Extended Data Fig. 7).

### TFM

For TFM^51^, gels were prepared and functionalized as described above, but the acrylamide mix was adjusted to yield a stiffness of roughly 400 Pa, as estimated by AFM. TFM gel mix: 550 µl of 7.6 mM HCL, 315.5 µl of double-distilled water (ddH_2_O), 0.5 µl of TEMED (Sigma), 30 µl of 2% *bis*-acrylamide (BioRad), 75 µl of 40% acrylamide (BioRad), 4 µl of 200 nm diameter beads resuspended at 0.2 µM (crimson beads, Invitrogen), 20 µl of 0.1 M NHS (*N*-hydroxysuccinimide, Sigma-Aldrich) and 5 µl of 10% ammonium persulfate (GE HealthCare). Once plated in these gels, cells were imaged for 25 min (with a frame rate of 2 min). Then, cells were removed by gently adding TrypLE (Gibco) for 10 min, and decellularized gels were imaged at the same rate for 25 min. Traction was then calculated by using a combination of built-in ImageJ and MATLAB based plugins and software^52^.

### Crysectioning

Embryos were fixed in a solution containing 4% formaldehyde, 0.25% Glutaraldehyde and 0.1% Tween-20, all dissolved in 1× PHEM buffer (60 mM PIPES, 25 mM HEPES, 10 mM EGTA and 4 mM MgSO_4_·7H_2_O). Fixation was overnight at 4 °C and then dehydrated in 100% methanol for at least 2 h at room temperature. Then the samples were rehydrated by using a battery of methanol/PBS 1× 75–50–25% washes, 10 min for each solution and finally incubated with PBS 1×. The embryos were then incubated twice for 15 min in 0.25% NaBH_4_/PBS w/v and washed with PBS1x. Then, embryos were embedded and oriented in a gelatine solution. Gelatine blocks were frozen at −80 °C in precooled isopentane. Samples were then sectioned in 20-μm slices using a cryostat (CM-3050S, Leica) and collected in SuperFrozen Slides (VWR International). The slides were dried overnight at room temperature and processed for immunostaining, as described below.

### Immunostaining in glass, hydrogels and cryosections

Fibronectin (mAb 4H2 anti-FN, DSHB)^53^ and acetylated α-Tubulin (T6793, Sigma-Aldrich)^54^ or E-cadherin (5D8, DSHB)^4,38,50^ were used for immunostaining in histological sections. To remove the gelatine after cryosectioning the samples were washed twice with PBS for 15 min at 37° and blocked for 2 h with 10% normal goat serum (NGS). Antibodies were diluted at 1:500 (anti-acet-α-Tubulin) and 1:1,000 (anti-fibronectin) in 10% NGS, incubated overnight at 4 °C and washed three times with 0.1% PBS-T (PBS, 0.1% Tween-20). Alexa-fluor (Thermo-Fisher) secondary antibodies were diluted 1:350 in 10% NGS with 1/400 DAPI (for nuclear staining). The samples were incubated in this mix overnight at 4 °C and washed three times with 0.1% PBS-T.

For acetylated α-Tubulin and α-Tubulin detection ex vivo, explants were fixed in Buffer PHEM 1× containing (4% formaldehyde; 0.25% glutaraldehyde; 0.1% Tween-20) for 10 min at room temperature, then subsequently treated with 0.25% NaBH_4_ in PBS w/v for 10 min and washed with PBS 1×; permeabilization was done with PBS 0.1% Triton X-100 for 7 min at room temperature. Then the explants were blocked with 10% NGS for 30 min. The primary antibody anti-acetylated α-Tubulin and anti-α-Tubulin^54^ were diluted at 1:500 and 1:1,000, respectively, in 10% NGS and incubated overnight at 4 °C. Explants were washed three times with PBS 0.1% Tween, incubated overnight at 4 °C with secondary antibody and diluted at 1:350 in 10% NGS. DAPI was diluted at 1:1,000 and mixed with the secondary antibodies.

Immunostaining on hydrogels proceeded as described above but the washes with agitation were replaced by rinses that were carefully performed (seven rinses each time). MOWIOL (EMD Millipore) was used as the mounting medium. Images were acquired as described below and fluorescence intensity was analysed using the measurement tool from ImageJ.

### Microscopy and time-lapse live imaging

#### Time-lapse imaging

Images for dispersion assays were acquired every 5 min at 18 °C using an upright microscope Zeiss Imager Z2/Apotome.2 equipped with a motorized stage and a camera (Hamamatsu Orca flash 4.0 v.2). A ×10 W objective (N-Achroplan ×10/0.3 M27 (FWD = 2.6 mm), Zeiss) was used.

#### In situ hybridization imaging and grafts

All images were captured at room temperature in agarose dishes containing PBS, using a dissecting microscope (MZ FL III, Leica) equipped with a camera (DFL420, Leica) and imaging software (IM50, Leica). Magnification was ×3.2.

#### Immunofluorescence imaging

Most of the images were acquired at room temperature using a Zeiss LSM980 system, equipped with two PMT and one GaAsPand, a ×40 W objective (C-Apochromat ×40/1.1 W Corr M27, Zeiss); same microscope, but a C-Apochromat ×25/1.515 oil immersion objective was used for the TFM experiments. Camera, filter wheels and shutters were controlled by Zeiss’s ZEN Blue v.3.0. Images in Extended Data Fig. 3 and Extended Data Fig. 6 were acquired with a Leica Stellaris 5 confocal system by using a ×63/1.4 oil immersion objective with ×1 or ×0.75 optical zoom, respectively. Images in Extended Data Fig. 7b were acquired in a Leica Thunder by using a HC PL APO ×20/0.80 PH2 objective. Camera, filter wheels and shutters were controlled by built-in Leica softwares.

### iAFM measurements

All AFM measurements were by using a FLEX-ANA (Nanosurf) automated AFM device, fitted with a *x*–*y* motorized stage and an automated software for experimental setup and analysis (ANA Software, Nanosurf).

#### In vivo AFM measurements

In our previous study we used cantilevers coated with a roughly 40 μm bead for in vivo AFM measurements of the mesoderm as a tissue^4^. Here, we used cantilevers coated with roughly 10 μm diameter colloidal spheres (CP-qp-SCONT-BSG-B-5, sQube). This tip size (10 μm) secured that our indentations capture the mechanical properties of the NC or the mesoderm and not the convolution of both. Cantilevers were mounted on the AFM device and their spring constants were calculated using the thermal noise method^55^. Only cantilevers with spring constants between 0.01 and 0.03 N m^−1^ were selected. Before use, we controlled that these cantilevers still capture tissue level stiffness (Extended Data Fig. 1; explained below in the section AFM tissue deformation control). Then, embryos were mounted in a plasticine dish and indentations were performed as described in Extended Data Fig. 1. The following modulation parameters were used: maximum indentation force, 10 nN; approach speed, 5 μm s^−1^; retraction speed was 55 μm s^−1^ and sample rate, 2,400 Hz.

For single cell AFM measurements in this experiment, the AFM head was set in a Leica Thunder inverted fluorescent microscope. This allowed to image cells with a HC PL APO ×20/0.80 PH2 objective while acquiring AFM data. A smaller cantilever coated with a roughly 2-μm diameter colloidal tip was used (CP-qp-SCONT-Au-A-5, sQube). After calculating their spring constant, 25 indentations were performed per cell in a region of interest of 10 × 10 μm^2^. The following modulation parameters were used: maximum indentation force, 2 nN; approach speed, 5 μm s^−1^; retraction speed, 55 μm s^−1^ and sample rate, 2,400 Hz.

### AFM tissue deformation control

To control that after reducing the cantilever bead size from 40 to 10 μm, we were still capturing the elastic properties of the NC as a collective, so we recorded the displacement of nRFP-labelled cell nuclei while applying a typical in vivo indentation. The same modulation parameters used for in vivo AFM measurements. Nuclei displacement on indentation was estimated with a built-in iterative particle image velocity ImageJ plugin and the same plugin was used to plot displacement maps.

### Data analysis and image treatment

#### AFM data analysis

In both cases, force–distance curves were fitted to a Hertz model for a spherical indenter,

$$F = \frac{4}{3}K\sqrt r \delta ^{3/2} = \frac{4}{3}\frac{E}{{1 - v^2}}\sqrt r \delta ^{3/2}$$

with applied force *F*, Young’s modulus *E*, Poisson’s ratio *v*, indenter radius *r*, indentation depth *δ* and apparent elastic moduli *K* = *E/*(1 – *ν*^2^), referred as ‘stiffness’ in the text and as ‘apparent elasticity (Pa)’ in the *y* axis of each chart. Force–distance curves were selected on the basis of their shape (example in Extended Data Fig. 1b)^56^. Then the apparent elastic moduli from in vivo AFM indentations were extracted at maximum indentation depth by using the built-in ANA AFM analysis software. Next, a 1 μm indentation depth was used to extract apparent elasticity from single cell indentations, as previously defined^57^. For single cell measurements, AtomicJ was used to identify the contact point and dissect 1 μm indentation depth. Then the median of each embryo or cell was calculated and processed for further statistical analyses.

#### In vivo analysis of NC migration

For in situ hybridization and grafted embryos, the length of the NC was obtained and normalized against the total dorso-ventral length of the embryo. Lengths were obtained using the built-in measurement tool from ImageJ and further analysed as described in Statistical analysis.

#### Ex vivo analysis of NC spreading

To extract the collective and individual dynamics from spreading NC clusters we used the squared radius of gyration (*R*_g_^2^), a suitable parameter to assess the trajectories and dynamics of migrating cells and clusters^16–18^. *R*_g_^2^ is an experimentally accessible output that allows for comparison of both simulations and ex vivo dispersion/spreading assays, and is defined as:

$$R_{\mathrm{g}}^2(t) = \frac{1}{{{{{N}}}}}{{{\mathrm{{\Sigma}}}}}_i^N\left( {{\bf r}_i(t) - {\bf r}_{{\mathrm{CM}}}(t)} \right)^2$$

where *N* is the number of cells, ${{\bf r}}_i(t)$ is the position of cell *i* at time *t* and ${{\bf r}}_{{\mathrm{CM}}} = \frac{1}{{{{{N}}}}}{{{\mathrm{{\Sigma}}}}}_i^N\,{{\bf r}}_i$ is the centre of mass of all cell positions. *R*_g_^2^ is the mean squared distance from the centre of the cell cluster and measures the average space that cells explore^17^. We also extracted the effectiveness of cell migration by quantifying the time dependence of *R*_g_^2^. By fitting the time dependence of radius of gyration squared to a power law function $R_{\mathrm{g}}^2(t)\approx t^\gamma$, we extracted the power exponent *γ*. An increase in radius of gyration squared with a power law exponent *γ* > 1 is indicative of efficient cell spreading compared to *γ* = 1, which is indicative of a random walk^18^ (inset in Fig. 1e). To extract the trajectory of cells from ex vivo experiments we used an ImageJ-based manual tracking plugin. Then these tracks were used to calculate and plot *R*_g_^2^ results by using custom made MATLAB codes. Data were further analysed as described in the Statistical analysis section.

### Image treatment

The *z*-stacks, maximum projections and time-lapse movies were created using ImageJ software. Adjustment of display map levels, re-sizing and addition of scale bars and pseudo colour were applied with ImageJ and/or Adobe Photoshop. In Fig. 4f the background was pseudo coloured in Adobe Illustrator.

### RNA-seq experiments and analyses

The quality of the extracted RNA was assessed using HS RNA Screen Tape Analysis (Agilent Technologies), libraries were generated by SMART-Seq2 and a Fragment Analyzer (AATI) was used for their quantification and to determine their quality. Libraries were then sequenced in a NextSeq500 Sequencer (Illumina) using a 75 SE high-throughput kit. Sequence information was extracted in FastQ format using the bcl2fastq v.2.19.1.403 (Illumina). Informatic analysis was carried out by the IGC Bioinformatics Unit by mapping the obtained sequences against the reference genome of *X. laevis*, version XENLA_9.2_Xenbase.gtf (v.9.2).

### Statistical analysis

No software was used for sample size determination. No randomization of the experiments was performed as, because of the nature of our experiments, only viable embryos and cell clusters were considered for analysis. Moreover, mis-injection was not included for in situ hybridization analysis meaning that the authors were not blinded to allocation while performing and/or analysing the experiments. For any of the mentioned cases, after selections, all parameters were measured at random.

Each experiment was repeated at least three times. Every set of data was tested for normality test using the, d’Agostino–Pearson and/or Shapiro–Wilk test in Prism7 (GraphPad). For paired comparisons, significances were calculated Prism7 with a Student’s *t*-test (two-tailed, unequal variances) when the distributions proved to be normal. If a data set did not pass the normality tests, the significances were calculated with Mann–Whitney (two-tailed, unequal variances). For multiple comparison of data with normal distribution unpaired one-way analysis of variance (ANOVA) with Bonferroni test correction was performed, while non-normal distribution data sets were analysed with Kruskal**–**Wallis corrected with Dunn’s test. Individual comparisons were calculated only when multiple comparisons showed *P* > 0.05 and significances in these cases were calculated in Prism7 as described for paired comparisons. The confidence interval in all experiments was 95% and as a detailed description of statistical parameters it is included in all figure captions.

### Reporting summary

Further information on research design is available in the Nature Research Reporting Summary linked to this article.

## Online content

Any methods, additional references, Nature Research reporting summaries, source data, extended data, supplementary information, acknowledgements, peer review information; details of author contributions and competing interests; and statements of data and code availability are available at 10.1038/s41563-022-01323-0.

## Supplementary information

Supplementary Information Supplementary Table 1, Note and references.

Reporting Summary

Supplementary Video 1 **Microtubule acetylation modulates cell migration** **ex vivo**. Ex vivo time-lapse of control, hyperacetylated and hypoacetylated neural crest cells migrating in a stiff substrate. Time-lapse setting was one picture every 6 min and 70 frames are shown.

Supplementary Video 2 **Cell autonomous effects of microtubule acetylation in NC migration** **ex vivo**. Ex vivo time-lapse of a mixture or mosaic made of control and hyperacetylated migrating in a stiff substrate. Time-lapse setting was one picture every 5 min and 140 frames are shown. Note that hyperacetylated cells (red) did not spread away from the cluster, unlike control cells.

Supplementary Video 3 **Piezo1 controls CCM via microtubules acetylation**. Ex vivo time-lapse of control, GsMTx4, Piezo1-MO and Piezo1-MO + HypoAcet neural crest cells migrating in a stiff substrate. Time-lapse setting was pme picture every 6 min and 70 frames are shown.

## Data Availability

The main data supporting the findings here are available with the paper. Source data used for *P* values are provided with this paper. Extra data and materials are available from the corresponding author upon reasonable request.

## Source data

Source Data Fig. 1 Source data.
Source Data Fig. 2 Source data.
Source Data Fig. 3 Source data.
Source Data Fig. 4 Source data.
Source Data Fig. 5 Source data.
Source Data Fig. 6 Source data.
Source Data Extended Data Fig. 1 Source data.
Source Data Extended Data Fig. 3 Source data.
Source Data Extended Data Fig. 6 Source data.
Source Data Extended Data Fig. 7 Source data.
Source Data Extended Data Fig. 8 Source data.
Source Data Extended Data Fig. 9 Source data.

## Extended data

are available for this paper at 10.1038/s41563-022-01323-0.

## Code availability

The custom codes used in the article are available at https://github.com/naseermk/ncmodeling.
