# Supplementary material for: Cell clusters softening triggers collective cell migration in vivo
Source: Nat Mater. 2022 Aug 15;21(11):1314–23. doi: 10.1038/s41563-022-01323-0 (PMC9622418; doi:10.1038/s41563-022-01323-0)
Supplement: Supplementary file 1 — Supplementary Table 1, Note and references. [file 41563_2022_1323_MOESM1_ESM.pdf]

nature portfolio
https://doi.org/10.1038/s41563-022-01323-0

Supplementary information

Cell clusters softening triggers collective cell migration in vivo

In the format provided by the authors and unedited

Supplementary Table:

Protein Family	Name	References
Piezo1	piezo type mechanosensitive ion channel component 1	[1]
Piezo2	piezo type mechanosensitive ion channel component 2	[1]
TRPA1	transient receptor potential cation channel subfamily A member 1	[2]
TRPV1	transient receptor potential cation channel subfamily V member 1	[3]
TRPV4	transient receptor potential cation channel subfamily V member 4	[4]

Supplementary Table 1: **RNA-seq data from isolated neural crest library**. While several molecules were found in our unbiassed screening, we selected just stretch activated channels that have been reported to mediate mechanosensing in other systems (see references). Next, we further filtered these candidates based on their predicted role in cell migration. Since Piezo1 fulfilled this criteria, we next focused in studying the role of Piezo1 in microtubule acetylation, cell mechanics and collective cell migration. Details in **Methods**.

Supplementary Note: computational modelling of cell mechanical response.

To evaluate whether cell mechanical response to microtubule (MT) acetylation facilitates collective cell migration (CCM) through cell-to-substrate stiffness mediated self-propulsion force we developed a three-dimensional active particle model using the agent-based framework. Such cell based off-lattice computational approach is known to effectively model how cell migration is impacted by cell properties such as its size, stiffness, and mechanical interaction with cell neighbours[5-9]. Individual cells are modelled as soft deformable spherical agents that interact with (i) other cells and with (ii) the substrate.

Cell dynamics

The net force, $\boldsymbol{F}_i$, on the ith cell is the vectorial sum of the forces experienced by a cell. We performed over damped (low Reynolds number[10]) dynamics without thermal noise because the viscosity is assumed to be large. Hence, the equation of motion for the ith cell is,

$$\dot{\boldsymbol{r}}_i=\frac{\boldsymbol{F}_i}{\gamma_i},$$

where r_i is the position of the ith cell centre, and γ_i is the friction coefficient. The forces experienced by a cell are described below.

Forces

Forces arising from cell-cell interaction, cell-substrate interaction and the active propulsion force arising from cell-to-substrate stiffness ratio are incorporated into the model. Cell-cell interaction consists of a soft repulsion term that limits spatial overlap between cells and an adhesive term accounting for cohesion between cells as mediated by cell-cell adhesion molecules. Cell-to-substrate interaction similarly accounts for a soft repulsive term that limits cell-substrate adhesion area and a cohesive term that tends to increase the adhesion area. In active particle models, a self-propulsive velocity term modelling the effect of self-generated forces in movement have been used in the context of Self Propelled Particle (SPP)[11-13] and Self Propelled Voronoi[14,15] models. While the self-propulsion term is important for modelling collective cell migration, its physical origin especially in view of the interplay between cell-substrate mechanical properties is unclear. In this context, we show that cell-to-substrate stiffness ratio is an important mediator of the self-propulsion force that cells generate to undergo migration.

Details of the force terms described above are provided below:

(i) Cell-cell interaction: The individual cells interact with other cells via short-ranged forces, consisting of elastic force (repulsion) and adhesive (attraction) force. The elastic force (F_{ij}^{el}) between two cells i and j of radio R_i and R_j is:

$$F_{ij}^{el} = \frac{h_{ij}^{\frac{3}{2}}}{\frac{3}{4}\left(\frac{1-\nu_i^2}{E_i} + \frac{1-\nu_j^2}{E_j}\right)\left(\frac{1}{R_i} + \frac{1}{R_j}\right)^{1/2}}$$

where ν_i and E_i are the Poisson ratio and elastic modulus of the ith cell and h_{ij} is the virtual overlap distance between the two cells. The adhesive force (F_{ij}^{ad}) is given by,

$$F_{ij}^{ad} = A_{ij} f^{ad} (\frac{1}{2})(c_i^{rec} c_j^{lig} + c_i^{lig} c_j^{rec})$$

where A_{ij} is the overlap area between the two interacting cells and f^{ad} determines the strength of the adhesive bond. We have normalized the receptor(rec) and ligand(lig) concentrations to satisfy $c_i^{rec} = c_j^{lig} = 0.9$. Cell-cell adhesion strength coefficient is fixed at $f^{ad} = 5 \times 10^{-6} \mu N/\mu m^2$ throughout the simulation.

(ii) Cell-substrate interaction: The cell-substrate elastic interaction (F_{sub}^{el}) is modelled based on the Hertz formalism:

$$F_{sub,i}^{el} = \frac{4}{3} \frac{R_i^{\frac{1}{2}} * \delta^{\frac{3}{2}}}{\left(\frac{1-\nu_{sub}^2}{E_{sub}} + \frac{1-\nu_i^2}{E_i}\right)}$$

where ν_{sub} and E_{sub} are the Poisson ratio and elastic modulus of the substrate and δ the indentation of the cell into the substrate.

The cell-substrate adhesive interaction is given by:

$$F_{sub,i}^{ad} = A_{sub,i} f_{sub}^{ad} (\frac{1}{2})(c_{sub}^{rec} c_i^{lig} + c_{sub}^{lig} c_i^{rec})$$

where $A_{sub,i}$ is the overlap area between the a cell and the substrate and f_{sub}^{ad} determines the strength of the cell-substrate adhesive bond. The substrate (sub) receptor (rec) and ligand(lig) concentrations are normalized to satisfy $c_{sub}^{rec} = c_{sub}^{lig} = 0.9$. Cell-substrate adhesion strength coefficient is set at $f_{sub}^{ad} = 9.25 \times 10^{-6} \mu N/\mu m^2$ for control cells, $f_{sub}^{ad} = 9.5 \times 10^{-6} \mu N/\mu m^2$ for hypoacetylated cells and $f_{sub}^{ad} = 9.0 \times 10^{-6} \mu N/\mu m^2$ for hyperacetylated cells. We assume that the adhesion co-efficients, receptor and ligand concentrations are constant as a function of time. As we are interested in the long-time limit of collective cell migratory behaviours (over 8 hrs), we work under the assumption that the short time fluctuations in these parameters are coarse-grained to constant values.

In addition to the mechanical interaction (elastic and adhesive forces) experienced by a cell, we incorporate a self-propulsion force F_i^p that depends on the ratio of the cell-to-substrate stiffness:

$$F_i^p = T_p \left(\frac{E_{sub}}{E_i}\right)^{\frac{7}{4}} \delta\, \widehat{\boldsymbol{p}_\iota}$$

where T_p is a propulsion force coefficient with units of tension (which we set to unity -1 $\mu N/\mu m$), δ the cell indentation into the substrate (as defined above) and the polarity vector $\boldsymbol{p}_i$ specifying the direction along which the propulsion force acts. The polarity vector is assigned randomly along $\widehat{\boldsymbol{p}_\iota} = (\sin(\phi)\cos(\theta), \sin(\phi)\sin(\theta), 0)$ where the polar angle ϕ is randomly chosen in the interval $[0, \frac{\pi}{2}]$ and the azimuthal angle θ picked randomly in the interval $[0,2\pi]$. We assume that the polarity vector is not correlated in time and changes randomly with time. The out of plane component of $\boldsymbol{p}_i$ is set to zero to ensure that cell remains in contact with the substrate. In the context of two-dimensional motion similar self-propulsion forces have been postulated[16,17]. The exponent $7/4$ was chosen as it gives good fit to experimentally observed time dependent cell migratory behaviours as quantified by cell and cluster spreading vs time. It is worth mentioning that slightly modifying the exponent to smaller or larger values lead to no change in the trends in which the conclusions that we report here are based (Data not shown).

Friction coefficient: There are two contributions to the friction co-efficient $\gamma_i = 6\pi\eta R_i + \gamma^{max}\Sigma_{j\ in\ NN(i)}\left(A_{ij}\frac{1}{2}\left(1+\frac{\vec{F}_i.\vec{n}_{ij}}{|\vec{F}_i|}\right)\times\frac{1}{2}\left(c_i^{rec}\,c_j^{lig} + c_j^{rec}c_i^{lig}\right)\right.$. The first term is the Stokes relation $(6\pi\eta R_i)$ which models the friction with the substrate. The second friction term takes into account adhesive friction depending on cell-to-cell contact surface area (A_{ij}), and receptor(ligand) concentrations $(c_i^{rec}(c_i^{lig}))$. The summation is over cell nearest neighbours $NN(i)$. For any cell i, an array with distances from all other cells to cell i is created. By calculating $R_i + R_j - |\vec{r_\iota} - \vec{r_j}|$ and sorting for cells j satisfying $R_i + R_j - |\vec{r_\iota} - \vec{r_j}| > 0$ (necessary for any cell j to be in contact with cell i we identify the nearest neighbors.

Simulation Details

In each simulation, we start with placing 20 cells in a three-dimensional (3D) domain of size $X\ x\ Yx\ Z = 32\mu m\ x\ 32\mu m\ x\ 15\mu m.$ The *X,Y,Z* positions are picked from a uniform random distribution with the bounds specified above. The margins of the *X,Y* domain can expand (free boundary) while the z-position of the cells are constrained to be on a fixed plane. In the initial 10 steps, we allow the cells to grow in size, divide or undergo death process to facilitate randomizing the positions between simulation runs. The details of these cell processes are described in our earlier works[6]. We do not allow cells to grow in size, divide or undergo death for the rest of the simulation for a total of 3000 steps based on which we compare simulation results to experiments as we do not observe cell division,

death etc during the experimental time frame. We use this scheme to randomize the initial conditions. The simulations are repeated at least 3 times per condition to ensure that initial conditions do not affect our conclusions. The time scale is assigned to 10 seconds per step (arbitrary units) to match the experimentally observed time scale of cell spreading. Codes are implemented in MATLAB.

In the simulation we model different cell stiffnesses corresponding to different levels of microtubule (MT) acetylation, keeping the substrate stiffness fixed at $E_{sub} = 150Pa$.

**Acetylation levels**	**Cell Stiffness (Pa)**
Hypo	75
Control	150
Hyper	400

For soft substrates, the substrate stiffness is reduced to $E_{sub} = 50Pa$. The extent of cell spreading in the simulation is quantified using radius of gyration squared R_g^2 as discussed in the Main Text.

Supplementary References:

1 Coste, B. *et al.* Piezo1 and Piezo2 are essential components of distinct mechanically activated cation channels. *Science* **330**, 55-60, doi:10.1126/science.1193270 (2010).

2 Corey, D. P. *et al.* TRPA1 is a candidate for the mechanosensitive transduction channel of vertebrate hair cells. *Nature* **432**, 723-730, doi:10.1038/nature03066 (2004).

3 Feng, N. H., Lee, H. H., Shiang, J. C. & Ma, M. C. Transient receptor potential vanilloid type 1 channels act as mechanoreceptors and cause substance P release and sensory activation in rat kidneys. *American journal of physiology. Renal physiology* **294**, F316-325, doi:10.1152/ajprenal.00308.2007 (2008).

4 Suzuki, M., Mizuno, A., Kodaira, K. & Imai, M. Impaired pressure sensation in mice lacking TRPV4. *The Journal of biological chemistry* **278**, 22664-22668, doi:10.1074/jbc.M302561200 (2003).

5 Drasdo, D. & Hohme, S. A single-cell-based model of tumor growth in vitro: monolayers and spheroids. *Physical biology* **2**, 133-147, doi:10.1088/1478-3975/2/3/001 (2005).

6 Malmi-Kakkada, A. N., Li, X., Samanta, H. S., Sinha, S. & Thirumalai, D. Cell Growth Rate Dictates the Onset of Glass to Fluidlike Transition and Long Time Superdiffusion in an Evolving Cell Colony. *Physical Review X* **8**, 021025, doi:10.1103/PhysRevX.8.021025 (2018).

7 Malmi-Kakkada, A. N., Li, X., Sinha, S. & Thirumalai, D. Dual Role of Cell-Cell Adhesion In Tumor Suppression and Proliferation Due to Collective Mechanosensing. *bioRxiv*, 683250, doi:10.1101/683250 (2020).

8 Schaller, G. & Meyer-Hermann, M. Multicellular tumor spheroid in an off-lattice Voronoi-Delaunay cell model. *Physical review. E, Statistical, nonlinear, and soft matter physics* **71**, 051910, doi:10.1103/PhysRevE.71.051910 (2005).

9 Sinha, S., Malmi-Kakkada, A. N., Li, X., Samanta, H. S. & Thirumalai, D. Spatially heterogeneous dynamics of cells in a growing tumor spheroid: comparison between theory and experiments. *Soft matter* **16**, 5294-5304, doi:10.1039/c9sm02277e (2020).

10 Purcell, E. M. Life at low Reynolds number. *American Journal of Physics* **45**, 3-11, doi:10.1119/1.10903 (1977).

11 Belmonte, J. M., Thomas, G. L., Brunnet, L. G., de Almeida, R. M. & Chate, H. Self-propelled particle model for cell-sorting phenomena. *Physical review letters* **100**, 248702, doi:10.1103/PhysRevLett.100.248702 (2008).

12 Shimoyama, N., Sugawara, K., Mizuguchi, T., Hayakawa, Y. & Sano, M. Collective motion in a system of motile elements. *Physical review letters* **76**, 3870-3873, doi:10.1103/PhysRevLett.76.3870 (1996).

13 Vicsek, T., Czirok, A., Ben-Jacob, E., Cohen, I. I. & Shochet, O. Novel type of phase transition in a system of self-driven particles. *Physical review letters* **75**, 1226-1229, doi:10.1103/PhysRevLett.75.1226 (1995).

14 Bi, D., Yang, X., Marchetti, M. C. & Manning, M. L. Motility-driven glass and jamming transitions in biological tissues. *Physical review. X* **6**, doi:10.1103/PhysRevX.6.021011 (2016).

15 Staddon, M. F. *et al.* Cooperation of dual modes of cell motility promotes epithelial stress relaxation to accelerate wound healing. *PLoS computational biology* **14**, e1006502, doi:10.1371/journal.pcbi.1006502 (2018).

16 Lin, S. Z., Ye, S., Xu, G. K., Li, B. & Feng, X. Q. Dynamic Migration Modes of Collective Cells. *Biophysical journal* **115**, 1826-1835, doi:10.1016/j.bpj.2018.09.010 (2018).

17 Smeets, B. *et al.* Emergent structures and dynamics of cell colonies by contact inhibition of locomotion. *Proceedings of the National Academy of Sciences of the United States of America* **113**, 14621-14626, doi:10.1073/pnas.1521151113 (2016).
