# Supplementary material for: Cell clusters softening triggers collective cell migration in vivo
Source: Nat Mater. 2022 Aug 15;21(11):1314–23. doi: 10.1038/s41563-022-01323-0 (PMC9622418; doi:10.1038/s41563-022-01323-0)
Supplement: Supplementary file 2 — Reporting Summary [file 41563_2022_1323_MOESM2_ESM.pdf]

nature portfolio

Corresponding author(s): Elias H Barriga, PhD

Last updated by author(s): May 2, 2022

Reporting Summary

Nature Portfolio wishes to improve the reproducibility of the work that we publish. This form provides structure for consistency and transparency in reporting. For further information on Nature Portfolio policies, see our Editorial Policies and the Editorial Policy Checklist.

Statistics

For all statistical analyses, confirm that the following items are present in the figure legend, table legend, main text, or Methods section.

n/a	Confirmed	
☐	☒	The exact sample size (*n*) for each experimental group/condition, given as a discrete number and unit of measurement
☐	☒	A statement on whether measurements were taken from distinct samples or whether the same sample was measured repeatedly
☐	☒	The statistical test(s) used AND whether they are one- or two-sided<br>*Only common tests should be described solely by name; describe more complex techniques in the Methods section.*
☒	☐	A description of all covariates tested
☐	☒	A description of any assumptions or corrections, such as tests of normality and adjustment for multiple comparisons
☐	☒	A full description of the statistical parameters including central tendency (e.g. means) or other basic estimates (e.g. regression coefficient) AND variation (e.g. standard deviation) or associated estimates of uncertainty (e.g. confidence intervals)
☐	☒	For null hypothesis testing, the test statistic (e.g. *F*, *t*, *r*) with confidence intervals, effect sizes, degrees of freedom and *P* value noted<br>*Give P values as exact values whenever suitable.*
☒	☐	For Bayesian analysis, information on the choice of priors and Markov chain Monte Carlo settings
☒	☐	For hierarchical and complex designs, identification of the appropriate level for tests and full reporting of outcomes
☐	☒	Estimates of effect sizes (e.g. Cohen's *d*, Pearson's *r*), indicating how they were calculated

Our web collection on statistics for biologists contains articles on many of the points above.

Software and code

Policy information about availability of computer code

Data collection	To acquire data we used the last updates available of LAS-X (V3.7.4 Leica), FLEXACAM Cl (former HMSO) (V1.11a Leica), Zen (Blue edition V3.4 Zeiss), Ana (V 1.3 Nanosurf).
Data analysis	The authors declare that custom codes used in the article are available in https://github.com/naseermk/ncmodeling. AtomicJ (V2.3.1) was used to analyse single cell AFM data. Fiji (ImageJ V2.0.0-rc-69/1.53k) was used to process image data. Then Excel (V16.54 2021) and Prism9 (V9.3.1) were used to process data for statistical analysis.

For manuscripts utilizing custom algorithms or software that are central to the research but not yet described in published literature, software must be made available to editors and reviewers. We strongly encourage code deposition in a community repository (e.g. GitHub). See the Nature Portfolio guidelines for submitting code & software for further information.

Data

Policy information about availability of data

All manuscripts must include a data availability statement. This statement should provide the following information, where applicable:

- Accession codes, unique identifiers, or web links for publicly available datasets
- A description of any restrictions on data availability
- For clinical datasets or third party data, please ensure that the statement adheres to our policy

The authors declare that the main data supporting their findings are available along the paper. Source data used for P values are provided with this paper. Extra data and materials are available from the corresponding author upon reasonable request.

Field-specific reporting

Please select the one below that is the best fit for your research. If you are not sure, read the appropriate sections before making your selection.

☒ Life sciences ☐ Behavioural & social sciences ☐ Ecological, evolutionary & environmental sciences

For a reference copy of the document with all sections, see nature.com/documents/nr-reporting-summary-flat.pdf

Life sciences study design

All studies must disclose on these points even when the disclosure is negative.

Sample size	No software was used for sample size determination. Specified in the Statistical analysis section of our methods.
Data exclusions	Inviable embryos and cells were excluded from our analyses. For in vivo analyses (graft and in situ hybridizations) embryos that were missinjected, determined by fluorescence intensity assessment, were also excluded from analyses. Specified in the Statistical analysis section of our methods.
Replication	All experiments were repeated 3 times, specified in each figure legend and in the Statistical analysis section of methods.
Randomization	Parameters for each experiment were allocated to experimental groups and measured at random. Specified in the Statistical analysis section of methods.
Blinding	Authors were not blinded because embryos and cells were selected prior analysis and the criteria for selection was correct delivery of the injected treatments and survival. Stated in the Statistical Analysis section of the methods.

Reporting for specific materials, systems and methods

We require information from authors about some types of materials, experimental systems and methods used in many studies. Here, indicate whether each material, system or method listed is relevant to your study. If you are not sure if a list item applies to your research, read the appropriate section before selecting a response.

Materials & experimental systems

n/a	Involved in the study	
☐	☒	Antibodies
☒	☐	Eukaryotic cell lines
☒	☐	Palaeontology and archaeology
☐	☒	Animals and other organisms
☒	☐	Human research participants
☒	☐	Clinical data
☒	☐	Dual use research of concern

Methods

n/a	Involved in the study	
☒	☐	ChIP-seq
☒	☐	Flow cytometry
☒	☐	MRI-based neuroimaging

Antibodies

Antibodies used	Anti-Fibronectin (mAb 4H2) DSHB. [5ug/ml].<br>Anti-acetylated alpha-Tubulin (T6793, Sigma Aldrich) [1/500].<br>Anti-alpha-Tubulin (DMIA (T6199), Sigma Aldrich) [1/1000].<br>anti-E-cadherin (5D8), DSHB [1/200].<br>goat anti-mouse Alexa Fluor 488 (Thermo Fisher Scientific, A-11001). [1/350].<br>goat anti-rabbit Alexa Fluor 647 (Thermo Fisher Scientific, A-21244). [1/350].<br>goat anti-rabbit Alexa Fluor 555 (Thermo Fisher Scientific, A-21429). [1/350].<br>goat anti-mouse Alexa Fluor 555 (Thermo Fisher Scientific, A-32728). [1/350].
Validation	anti-Fibronectin was validated in Alfandari et al 2003, Dev Biol.<br>anti-acetylated alpha-Tubulin and anti-alpha-Tubulin were validated in Brooks and Wallingford 2015, Meth Cell Biol. anti-E-cadherin was validated in Nandadasa et al, 2009, Development.<br>Secondary antibodies were commercially validated and have been widely used in Xenopus laevis (i.e., Barriga et al., 2018, Nature; Barriga et al 2013, JCB; Shellard et al., 2021, Nature, etc):<br>goat anti-mouse Alexa Fluor 488 (Thermo Fisher Scientific, A-11001). [1/350].<br>goat anti-rabbit Alexa Fluor 647 (Thermo Fisher Scientific, A-21244). [1/350].<br>goat anti-rabbit Alexa Fluor 555 (Thermo Fisher Scientific, A-21429). [1/350].<br>goat anti-mouse Alexa Fluor 555 (Thermo Fisher Scientific, A-32728). [1/350].

Animals and other organisms

Policy information about studies involving animals; ARRIVE guidelines recommended for reporting animal research

Laboratory animals	Adults female (Xenopus laevis were subjected to hyperovulation protocols to obtain oocytes and these oocytes were then fertilised with a mix of sperms from adult males. Adult animals were aged 2 to 5 years. We analysed embryos at Xenopus neurula stages 13, 17 and 23 (defined as non-migratory, pre-migratory, and migratory stages, respectively).
Wild animals	The study did not involved wild animals.
Field-collected samples	The study did not involved field-collected samples.
Ethics oversight	All animal experiments were approved by Ethics Committee and the Animal Welfare Body of the IGC and by the Direcao Geral de Alimentacao e Veterinaria (DGAV).

Note that full information on the approval of the study protocol must also be provided in the manuscript.
